# Assessment of haemoglobin and serum markers of iron deficiency in people with cardiovascular disease

**DOI:** 10.1136/heartjnl-2022-322145

**Published:** 2023-05-02

**Authors:** Fraser J Graham, Jocelyn M Friday, Pierpaolo Pellicori, Nicola Greenlaw, John GF Cleland

**Affiliations:** 1 Robertson Centre for Biostatistics and Clinical Trials, University of Glasgow, Glasgow, UK; 2 School of Cardiovascular and Metabolic Health, University of Glasgow, Glasgow, UK

**Keywords:** biomarkers, epidemiology, electronic health records, heart failure

## Abstract

**Background:**

The prevalence of anaemia and iron deficiency and their prognostic association with cardiovascular disease have rarely been explored at population level.

**Methods:**

National Health Service records of the Greater Glasgow region for patients aged ≥50 years with a broad range of cardiovascular diagnoses were obtained. During 2013/14, prevalent disease was identified and results of investigations collated. Anaemia was defined as haemoglobin <13 g/dL for men or <12 g/dL for women. Incident heart failure, cancer and death between 2015 and 2018 were identified.

**Results:**

The 2013/14 dataset comprised 197 152 patients, including 14 335 (7%) with heart failure. Most (78%) patients had haemoglobin measured, especially those with heart failure (90%). Of those tested, anaemia was common both in patients without (29%) and with heart failure (prevalent cases in 2013/14: 46%; incident cases during 2013/14: 57%). Ferritin was usually measured only when haemoglobin was markedly depressed; transferrin saturation (TSAT) even less often. Incidence rates for heart failure and cancer during 2015–18 were inversely related to nadir haemoglobin in 2013/14. A haemoglobin of 13–15 g/dL for women and 14–16 g/dL for men was associated with the lowest mortality. Low ferritin was associated with a better prognosis and low TSAT with a worse prognosis.

**Conclusion:**

In patients with a broad range of cardiovascular disorders, haemoglobin is often measured but, unless anaemia is severe, markers of iron deficiency are usually not. Low haemoglobin and TSAT, but not low ferritin, are associated with a worse prognosis. The nadir of risk occurs at haemoglobin 1–3 g/dL above the WHO definition of anaemia.

**WHAT IS ALREADY KNOWN ON THIS TOPIC**

- The current definition of anaemia commonly used in cardiovascular research and clinical practice is based on a haemoglobin concentration below the fifth percentile of age and sex in mostly young, healthy individuals rather than on its association with clinical outcomes.

**WHAT THIS STUDY ADDS**

- The current definition of anaemia in patients with cardiovascular disease, with or without heart failure, misses a substantial proportion of patients at increased risk of heart failure, cancer or death.
- Haemoglobin is usually measured but, even in the presence of profound anaemia, iron indices are not.

**HOW THIS STUDY MIGHT AFFECT RESEARCH, PRACTICE OR POLICY**

- Clinicians should consider investigating patients with cardiovascular disease and borderline anaemia (haemoglobin 0–1 g/dL above the WHO definition) for iron deficiency.
- Future research should consider challenging the definition of anaemia in patients with cardiovascular disease.

## Introduction

In the general population, both low and very high concentrations of haemoglobin are associated with greater morbidity and mortality.^1 2^ Very high concentrations of haemoglobin are infrequent, and usually reflect smoking habits, chronic lung disease or, more rarely, myeloproliferative disorders. Low concentrations of haemoglobin are much more common. Although anaemia might be due to physiological blood loss in premenopausal women, in older individuals it is often a marker of comorbid conditions or their treatments, which reduce erythropoiesis, or predispose to malabsorption and/or blood loss.^3 4^ The most common cause of anaemia worldwide is iron deficiency,^5^ for which treatment is readily available, but it is unclear how often cases of anaemia are investigated for iron deficiency and which tests are most commonly used.

Although widely used for epidemiological studies, the definition of anaemia suggested by WHO is based on research conducted >50 years ago on young, otherwise healthy individuals; therefore, it should be extrapolated with caution to contemporary clinical practice for patients with cardiovascular disease.^6^ Electronic health records (EHR) provide routinely collected data to address important research questions and audit quality of care in large populations managed in clinical practice.

Accordingly, we used de-identified data to investigate the distribution of haemoglobin concentrations and their associations with outcome in a large cohort of adults with a broad range of cardiovascular diseases, including hypertension, atherosclerotic disease and heart failure. We also assessed how often diagnostic investigations for anaemia and iron deficiency were done, and the link between haemoglobin concentrations and subsequent incidence of cancer, heart failure and death.

## Methods

### Study population

The Glasgow SafeHaven links and provides secure, de-identified, routinely collected EHR for people managed by NHS Greater Glasgow and Clyde; a population of approximately 1.1 million in 2012. Linked data include demographics, blood tests (from primary and secondary care), electrocardiography, community prescription records, hospital admissions and related diagnoses and deaths.

For this analysis, we obtained authorisation for access to de-identified patient information for adults aged ≥50 years who, between 1 January 2010 and 1 April 2018, had a new or existing diagnosis of coronary or peripheral arterial disease or heart failure or with repeated prescriptions of treatments such as ACE inhibitors (ACEi), angiotensin II receptor blockers (ARBs), beta-blockers, mineralocorticoid receptor antagonists or loop diuretics (online supplemental tables S1–S5). These criteria were designed to capture a broad range of cardiovascular problems. Data from 2010 to 2012 were used to provide a medical history and identify prevalent anaemia. Patients with <12 months of data were excluded as were patients with end-stage renal disease, defined as an estimated glomerular filtration rate (eGFR) of <20 mL/min/1.73 m^2^, chronic kidney disease stage 5 or on renal dialysis, as such patients are already known to have a high prevalence of anaemia and a poor prognosis.

10.1136/heartjnl-2022-322145.supp1 Supplementary data

From 1 January 2013 to 31 December 2014 (testing period), we collated blood test results to identify how often haemoglobin, ferritin and transferrin saturations (TSAT) were measured. Most measurements were taken by primary care physicians or at outpatient clinics. Measurements of haemoglobin obtained during admissions for gastrointestinal haemorrhage or those due to trauma were excluded. Otherwise, only the first blood test during a hospital admission was used for this analysis to avoid confounding due to blood loss from surgery or other procedures. The nadir value for each test during this period was used for analyses, assuming that low values might act as a trigger for a therapeutic response designed to correct the abnormality.

Patients were also classified according to a known diagnosis of heart failure prior to 1 January 2013 (prevalent heart failure), incident heart failure between 1 January 2013 and 31 December 2014 and no recorded diagnosis of heart failure prior to 31 December 2014.

Patients within each diagnostic group were then stratified according to haemoglobin concentration into seven groups relative to the WHO definition: severe anaemia (>2 g/dL below); moderate anaemia (1–2 g/dL below); mild anaemia (0–1 g/dL below); borderline (>0–1 g/dL above), >1–3 g/dL above, >3–4 g/dL above and >4 g/dL above the WHO definition. Four definitions of iron deficiency were considered: serum ferritin <30 µg/L; serum ferritin <100 µg/L, serum iron ≤13 µmol/L and TSAT <20%.^7^

From 2015 to 2018, patients were followed to identify incident cases of heart failure and cancer and mortality, including causes of death.

### Statistics

Descriptive data are shown as numbers and percentage when categorical and as median with first and third quartiles if continuous. Mortality from 1 January 2015 until 31 March 2018 (last day of follow-up) was reported for patients according to heart failure diagnosis and nadir of haemoglobin or iron deficiency categories described above. All multivariable Cox models were adjusted for age and sex. No imputation was performed for missing data. Associations between haemoglobin and mortality are presented using Kaplan-Meier survival curves and/or forest plots. Associations between rates of retesting of haemoglobin in those with severe anaemia and between haemoglobin and incident heart failure diagnoses were analysed using competing risk models and presented in cumulative events curves with death as a competing risk. Patient groups with high haemoglobin concentrations (>3 g/dL above the WHO definition) were combined in some mortality analyses due to small patient numbers. Rates of testing of iron indices by age and sex are compared by χ^2^ tests. All statistical analysis was conducted with ‘R’ V.4.0.5 (supplements).

### Patient and public involvement

Patients and/or the public were not involved in the design, or conduct, or reporting, or dissemination plans of this research.

## Results

From an initial population of 364 785 individuals, after excluding mislinked data (n=1176), those aged <50 years (n=123 143) or censored before 1 January 2013 (n=21 844), those with missing data (n=5098) and those with end-stage renal disease (n=16 372), 1 97 152 patients were included in this analysis (online supplemental figure S1).

Prior to 2013, 10 678 (5%) patients were reported to have heart failure and a further 3657 (2%) developed heart failure in 2013/14. Patients with heart failure were older, more likely to be men and more likely to have ischaemic heart disease (IHD), diabetes, hypertension, atrial fibrillation, chronic obstructive airways disease and have a lower eGFR (table 1).

**Table 1:** Characteristics according to the presence (prevalent or incident) or absence of heart failure during 2013/14

	Not heart failure		Incident heart failure		Prevalent heart failure	
Demographics and comorbidities on or before 1 January 2013 unless stated otherwise						
	Surviving at 31 December 2014	Died before 31 December 2014	Surviving at 31 December 2014	Died before 31 December 2014	Surviving at 31 December 2014	Died before 31 December 2014
N (%)	172 940	9877	2776	881	8899	1779
Age (years)	65 (58–74)	79 (70–86)	74 (65–81)	82 (75–87)	72 (64–80)	82 (75–88)
Sex (women)	95 280 (55%)	5351 (54%)	1291 (47%)	452 (51%)	3478 (39%)	903 (51%)
Hypertension	57 633 (33%)	3009 (30%)	1281 (46%)	281 (32%)	4848 (54%)	851 (48%)
Diabetes or hypoglycaemic therapy	28 244 (16%)	1767 (18%)	553 (20%)	156 (18%)	2122 (24%)	421 (24%)
IHD	32 400 (19%)	2556 (26%)	984 (35%)	291 (33%)	6568 (74%)	1151 (65%)
COPD	17 168 (10%)	1894 (19%)	531 (19%)	169 (19%)	2035 (23%)	588 (33%)
eGFR (last available prior to 2013)	82 (71–94)	75 (59–91)	78 (65–91)	72 (58–86)	75 (61–90)	65 (49–82)
eGFR available	154 816 (90%)	9383 (95%)	2499 (90%)	841 (95%)	8690 (98%)	1755 (99%)
GI disease	5360 (3%)	907 (9%)	130 (5%)	60 (7%)	462 (5%)	167 (9%)
Any cancer prior to 2013	10 780 (6%)	2127 (22%)	216 (8%)	123 (14%)	756 (8%)	327 (18%)
Any incident cancer 2013/14	3376 (2%)	2119 (21%)	116 (4%)	114 (13%)	215 (2%)	208 (12%)
ECG (last result available between 2010 and 31 December 2014)						
ECG available	49 022 (28%)	4534 (46%)	1992 (72%)	549 (62%)	4010 (45%)	917 (52%)
AF/Flutter	3890 (8%)	848 (19%)	588 (30%)	169 (31%)	961 (24%)	314 (34%)
Haemoglobin results						
Test prior to 2013 (yes/no)	137 812 (80%)	9047 (92%)	2339 (84%)	811 (92%)	8195 (92%)	1719 (97%)
Anaemia prior to 2013 (% of those tested)	37 828 (27%)	5450 (60%)	959 (41%)	468 (58%)	3714 (45%)	1235 (72%)
Test during 2013/14 (yes/no)	132 200 (76%)	8411 (85%)	2704 (97%)	873 (99%)	7806 (88%)	1515 (85%)
Anaemia 2013/14 (% of those tested)	35 310 (27%)	5651 (67%)	1418 (52%)	604 (69%)	3265 (42%)	1051 (69%)
Incident anaemia 2013/14	13 992 (11%)	1825 (22%)	666 (25%)	210 (24%)	887 (11%)	197 (13%)
Hb (median/quartiles)	13.3 (12.2–14.4)	11.4 (9.8–12.9)	12.3 (10.7–13.6)	11.2 (9.7–12.7)	12.9 (11.5–14.2)	11.4 (9.8–12.8)
Prescriptions (anytime in 2013 or 2014)						
Iron (oral)	13 817 (8%)	1750 (18%)	584 (21%)	233 (26%)	1377 (15%)	435 (24%)
B_12_	7689 (4%)	680 (7%)	203 (7%)	71 (8%)	593 (7%)	132 (7%)
Folate	12 137 (7%)	1601 (16%)	365 (13%)	172 (20%)	1028 (12%)	312 (18%)
Loop diuretics	21 431 (12%)	3517 (36%)	1814 (65%)	551 (63%)	4744 (53%)	1255 (71%)
ACEi/ARB	94 839 (55%)	4089 (41%)	2161 (78%)	468 (53%)	7186 (81%)	965 (54%)
BB	64 274 (37%)	3529 (36%)	2005 (72%)	401 (46%)	6392 (72%)	906 (51%)
MRA	1737 (1%)	391 (4%)	511 (18%)	77 (9%)	1216 (14%)	255 (14%)
Antiplatelets	66 090 (38%)	5021 (51%)	2015 (73%)	547 (62%)	6238 (70%)	1124 (63%)
OAC	9639 (6%)	822 (8%)	897 (32%)	164 (19%)	2396 (27%)	387 (22%)
NSAID	48 991 (28%)	1232 (12%)	524 (19%)	90 (10%)	1201 (13%)	95 (5%)
Insulin	4115 (2%)	309 (3%)	108 (4%)	33 (4%)	426 (5%)	94 (5%)
Other hypoglycaemic agents	23 015 (13%)	1293 (13%)	470 (17%)	119 (14%)	1625 (18%)	258 (15%)
PPI/H2 antagonist	87 992 (51%)	5939 (60%)	1799 (65%)	547 (62%)	5447 (61%)	1151 (65%)
Deaths 2013–2014						
Age at death	NA	80 (71–87)	NA	83 (76–88)	NA	83 (76–89)
All	0 (0%)	9877 (100%)	0 (0%)	881 (100%)	0 (0%)	1779 (100%)
Cancer	NA	3288 (33%)	NA	118 (13%)	NA	306 (17%)
GI cancer	NA	877 (9%)	NA	28 (3%)	NA	77 (4%)
CVD	NA	2666 (27%)	NA	401 (46%)	NA	755 (42%)
Neurological	NA	1087 (11%)	NA	37 (4%)	NA	116 (7%)
Chronic respiratory	NA	1018 (10%)	NA	123 (14%)	NA	244 (14%)
Infection	NA	799 (8%)	NA	104 (12%)	NA	181 (10%)
Other	NA	1019 (10%)	NA	98 (11%)	NA	177 (10%)

Presented as number and (%) or median and (Q1–Q3) for continuous variables.

Sodium-glucose co-transporter 2 inhibitors were used in ≤1% of individuals in all categories.

Blood results reported are the nadir result unless otherwise stated. The presence of any of anaemia or AF/flutter is reported.

ACEi/ARB, ACE inhibitor/angiotensin receptor blocker; AF, atrial fibrillation; BB, beta-blocker; COPD, chronic obstructive pulmonary disease; CVD, cardiovascular disease; eGFR, estimated glomerular filtration rate; GI, gastrointestinal; IHD, ischaemic heart disease; MRA, mineralocorticoid receptor antagonist; NA, not available; NSAID, non-steroidal anti-inflammatory drugs; OAC, oral anticoagulant; PPI, proton pump inhibitors.

### Testing patterns and results of testing

Most patients had haemoglobin measured both before and between 2013 and 2014. Patients with heart failure were more likely to be tested and more likely to have anaemia (table 1). Most of those with anaemia in 2013/14 already had anaemia prior to 2013, and >1 in 10 developed new-onset anaemia between 2013 and 2014; new-onset anaemia was common (25%) in those newly diagnosed with heart failure during this period. Of those without anaemia prior to 2013, those closest to the WHO threshold (haemoglobin 0–1 g/dL above) were most at risk of developing anaemia (online supplemental tables S6–S8). In those with severe anaemia (>2 g/dL below the WHO definition) identified in an outpatient setting during 2013/14, rates of subsequent retesting of haemoglobin were high, with >50% retested within 1 month irrespective of heart failure group (online supplemental figures S2–S4).

**Figure 1:**
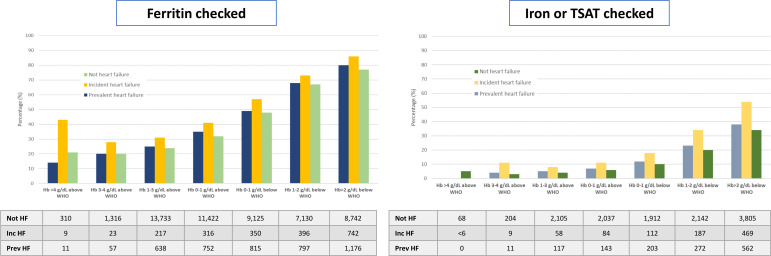
Testing patterns of iron biomarkers according to haemoglobin concentration. Ferritin on the left and, separately on the right, serum iron or transferrin saturation (TSAT) according to haemoglobin concentrations and patient group (not heart failure (HF); incident HF; prevalent HF). Numbers are presented below each graph.

Rates of testing of iron indices increased as severity of anaemia increased, with around 80% of those with haemoglobin >2 g/dL below the WHO definition having at least one test for iron deficiency (figure 1). Serum iron or TSAT were measured much less frequently (8% of all with haemoglobin measured; 20% if anaemia) than ferritin (38% of all with haemoglobin measured; 62% if anaemia). In general, women and those aged >70 years were more likely to have iron tests (online supplemental tables S9 and S10) and they were done slightly more often among those with incident heart failure compared with other patients.

When investigated, iron deficiency (by all definitions) was more common as haemoglobin decreased (table 2). Of those with anaemia investigated for iron deficiency (table 2), >50% had a ferritin <100 µg/L, a serum iron ≤13 µmol/L and a TSAT <20%. A large proportion of those without anaemia, even when haemoglobin was >1 g/dL above the WHO definition, also had one or more blood tests suggesting iron deficiency.

**Table 2:** Haematology profile and iron measurements according to HF group during 2013/14

	Hb by WHO grade	N	F done (yes vs no)	F <30 µg/L (% of all/% of those tested)	F <100 µg/L (% of all/% of those tested)	S. iron done (yes vs no)	S. iron ≤13 µmol/L (% of all/% of those tested)	TSAT done (yes vs no)	TSAT <20 % (% of all/% of those tested)	MCV <80 fl
Not HF	Not done	42 206 (23%)	184 (0%)	16 (<1%/9%)	82 (<1%/45%)	39 (0%)	19 (<1%/49%)	38 (0%)	11 (<1%/29%)	0 (NA%)
	>4	1447 (1%)	310 (21%)	13 (1%/4%)	93 (6%/30%)	68 (5%)	14 (1%/21%)	67 (5%)	10 (1%/15%)	6 (0%)
	>3 to 4	6469 (4%)	1316 (20%)	59 (1%/4%)	480 (7%/36%)	204 (3%)	47 (1%/23%)	204 (3%)	40 (1%/20%)	29 (0%)
	>1 to 3	56 341 (31%)	13 733 (24%)	1370 (2%/10%)	7006 (12%/51%)	2105 (4%)	691 (1%/33%)	2085 (4%)	593 (1%/28%)	596 (1%)
	≥0 to 1	35 393 (19%)	11 422 (32%)	2087 (6%/18%)	6968 (20%/61%)	2037 (6%)	1002 (3%/49%)	2018 (6%)	838 (2%/42%)	971 (3%)
	<0 to 1	18 938 (10%)	9125 (48%)	2486 (13%/27%)	5902 (31%/65%)	1912 (10%)	1249 (7%/65%)	1900 (10%)	1060 (6%/56%)	1226 (6%)
	<−1 to 2	10 706 (6%)	7130 (67%)	2492 (23%/35%)	4753 (44%/67%)	2142 (20%)	1655 (15%/77%)	2128 (20%)	1436 (13%/67%)	1303 (12%)
	<−2	11 317 (6%)	8742 (77%)	3554 (31%/41%)	5692 (50%/65%)	3805 (34%)	3129 (28%/82%)	3789 (33%)	2778 (25%/73%)	3018 (27%)
Incident HF	Not done	80 (2%)	<6 (NA%)	0 (0%/0%)	0 (0%/0%)	0 (0%)	0 (NA%/NA%)	0 (0%)	0 (0%/0%)	0 (NA%)
	>4	21 (1%)	9 (43%)	0 (0%/0%)	<6 (NA%/NA%)	<6 (NA%)	<6 (NA%/NA%)	<6 (NA%)	<6 (NA%/NA%)	0 (0%)
	>3 to 4	83 (2%)	23 (28%)	0 (0%/0%)	<6 (NA%/NA%)	9 (11%)	<6 (NA%/NA%)	9 (11%)	<6 (NA%/NA%)	<6 (NA%)
	>1 to 3	689 (19%)	217 (31%)	10 (1%/5%)	87 (13%/40%)	58 (8%)	34 (5%/59%)	58 (8%)	33 (5%/57%)	11 (2%)
	≥0 to 1	762 (21%)	316 (41%)	46 (6%/15%)	166 (22%/53%)	84 (11%)	59 (7%/70%)	84 (11%)	54 (7%/64%)	35 (5%)
	<0 to 1	614 (17%)	350 (57%)	82 (13%/23%)	191 (31%/55%)	112 (18%)	95 (15%/85%)	110 (18%)	77 (13%/70%)	56 (9%)
	<−1 to 2	545 (15%)	396 (73%)	93 (17%/23%)	242 (44%/61%)	187 (34%)	162 (30%/87%)	186 (34%)	146 (27%/78%)	65 (12%)
	<−2	863 (24%)	742 (86%)	234 (27%/32%)	489 (57%/66%)	469 (54%)	417 (48%/89%)	467 (54%)	383 (44%/82%)	228 (26%)
Prevalent HF	Not done	1357 (13%)	16 (1%)	<6 (NA%/NA%)	<6 (NA%/NA%)	7 (1%)	<6 (NA%/NA%)	7 (1%)	<6 (NA%/NA%)	0 (NA%)
	>4	78 (1%)	11 (14%)	0 (0%/0%)	<6 (NA%/NA%)	0 (0%)	0 (NA%/NA%)	0 (0%)	0 (0%/0%)	0 (0%)
	>3 to 4	284 (3%)	57 (20%)	<6 (NA%/NA%)	23 (8%/40%)	11 (4%)	<6 (NA%/NA%)	11 (4%)	<6 (NA%/NA%)	<6 (NA%)
	>1 to 3	2503 (23%)	638 (25%)	43 (2%/7%)	287 (11%/45%)	117 (5%)	58 (2%/50%)	116 (5%)	46 (2%/40%)	29 (1%)
	≥0 to 1	2140 (20%)	752 (35%)	103 (5%/14%)	394 (18%/52%)	143 (7%)	87 (4%/61%)	142 (7%)	71 (3%/50%)	63 (3%)
	<0 to −1	1675 (16%)	815 (49%)	181 (11%/22%)	470 (28%/58%)	203 (12%)	134 (8%/66%)	203 (12%)	119 (7%/59%)	93 (6%)
	<−1 to −2	1180 (11%)	797 (68%)	226 (19%/28%)	500 (42%/63%)	272 (23%)	213 (18%/78%)	270 (23%)	181 (15%/67%)	119 (10%)
	<−2	1461 (14%)	1176 (80%)	404 (28%/34%)	766 (52%/65%)	562 (38%)	488 (33%/87%)	561 (38%)	435 (30%/78%)	346 (24%)

F, ferritin; Hb, haemoglobin; HF, heart failure; MCV, mean cell volume; NA, not available; S., serum; TSAT, transferrin saturation.

### Associations between haemoglobin concentrations, treatments and incident cancer or heart failure

Patients with lower haemoglobin concentrations were older and more likely to have diabetes, IHD and gastrointestinal (GI) diseases, and a lower eGFR, than those without. Prescriptions of oral iron, folate and B12 therapies, loop diuretics and particularly proton pump inhibitors (PPIs)/H2-receptor antagonists were higher among patients with lower haemoglobin concentrations. For all patients with heart failure, those with lower haemoglobin concentrations were less likely to receive beta-blockers and ACEi or ARBs (online supplemental tables S6–S8).

An inverse relation was present between rates of both prevalent and incident cancer diagnoses and haemoglobin concentration in patients with or without heart failure (online supplemental tables S6–S8). Between 2013 and 2014, rates of incident cancer were highest in those with severe anaemia (7%–11%).

In those without prior heart failure, a non-linear relationship was present between haemoglobin and rates of new-onset heart failure (after 1 January 2015) (online supplemental figure S5): those with haemoglobin concentrations >2 g/dL below the WHO definition of anaemia had the highest risk of developing heart failure, despite a higher mortality as a competing risk.

### Associations with mortality

Of 197 152 patients in this analysis, 12 537 died between 1 January 2013 and 31 December 2014. Rates of death were higher for those with incident (24%) or prevalent (17%) heart failure compared with those without heart failure (5%) (table 1).

From 2015 onwards, of those with cardiovascular disease but not heart failure 11% died compared with 25% of those with heart failure (online supplemental tables S6–S8). Most patients with heart failure died from cardiovascular causes irrespective of haemoglobin concentration. Cancer (n=5313; 29% of deaths) and cardiovascular events (n=4884; 27% of deaths) were similarly common causes of death in patients without heart failure.

**Figure 2:**
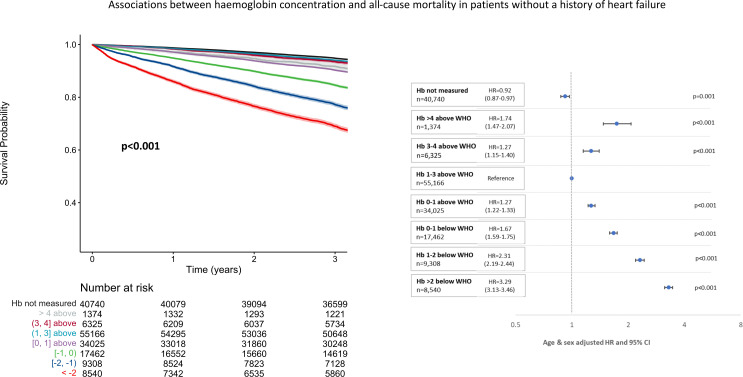
Unadjusted Kaplan-Meier survival curves and corresponding forest plots showing associations between haemoglobin (Hb) concentrations and mortality from 1 January 2015 to 31 March 2018 in patients without heart failure recorded at any time. Numbers at risk presented with each Kaplan-Meier and age-adjusted and sex-adjusted HRs with corresponding 95% CIs also presented.

**Figure 3:**
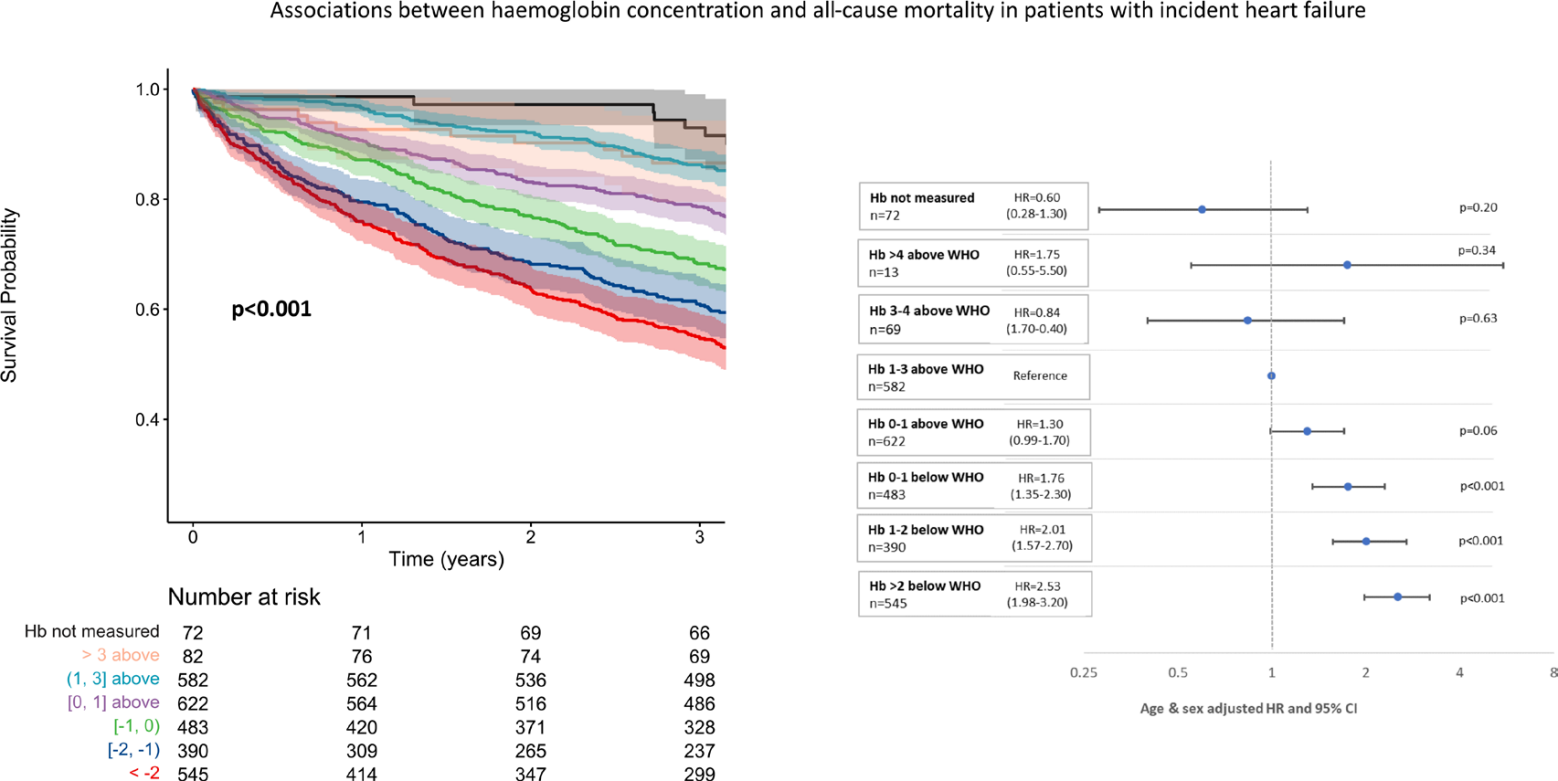
Unadjusted Kaplan-Meier survival curves and corresponding forest plots showing associations between haemoglobin (Hb) concentrations and mortality from 1 January 2015 to 31 March 2018 in patients with incident heart failure between 1 January 2013 and 31 December 2014. Numbers at risk presented with each Kaplan-Meier and age-adjusted and sex-adjusted HRs with corresponding 95% CIs also presented.

**Figure 4:**
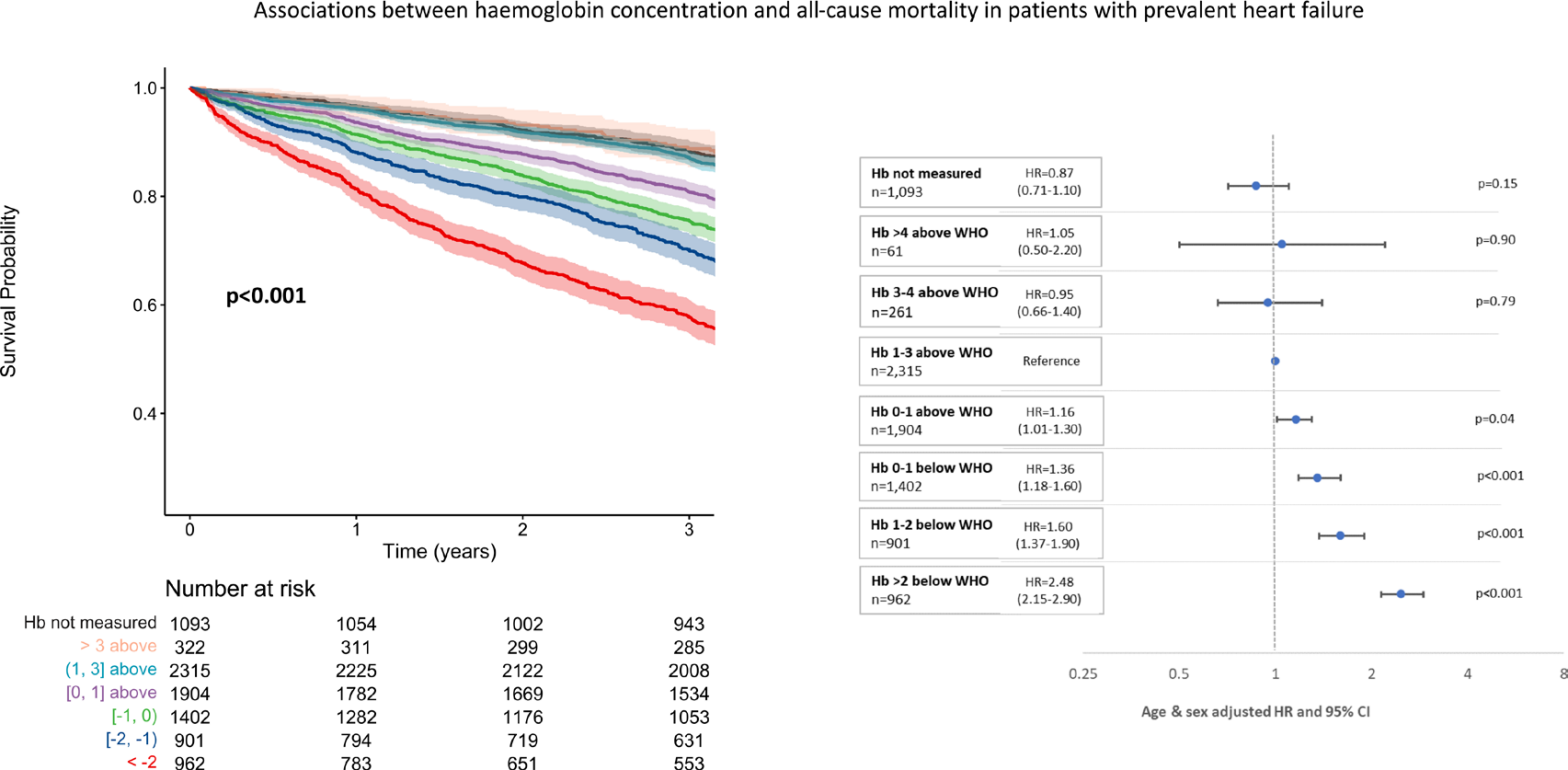
Unadjusted Kaplan-Meier survival curves and corresponding forest plots showing associations between haemoglobin (Hb) concentrations and mortality from 1 January 2015 to 31 March 2018 in patients with prevalent heart failure prior to 1 January 2013. Numbers at risk presented with each Kaplan-Meier and age-adjusted and sex-adjusted HRs with corresponding 95% CIs also presented.

Of 184 615 patients alive at the end of the 2013/14 testing period, those in whom haemoglobin had not been measured had the best and those with severe anaemia (>2 g/dL below the WHO definition) the worst outcomes across all diagnostic groups (figures 2–4). There was a U-shaped relationship between haemoglobin and all-cause mortality, particularly in those without a history of heart failure. Compared with those with a haemoglobin of 1–3 g/dL above the WHO definition of anaemia, mortality was greater both for those with borderline anaemia (haemoglobin 0–1 g/dL above the WHO definition) and those with a haemoglobin >3 g/dL above the WHO definition. Patients with higher haemoglobin had a greater proportion of deaths due to chronic respiratory diseases compared with those with normal or low haemoglobin concentrations (online supplemental tables S6–S8).

**Figure 5:**
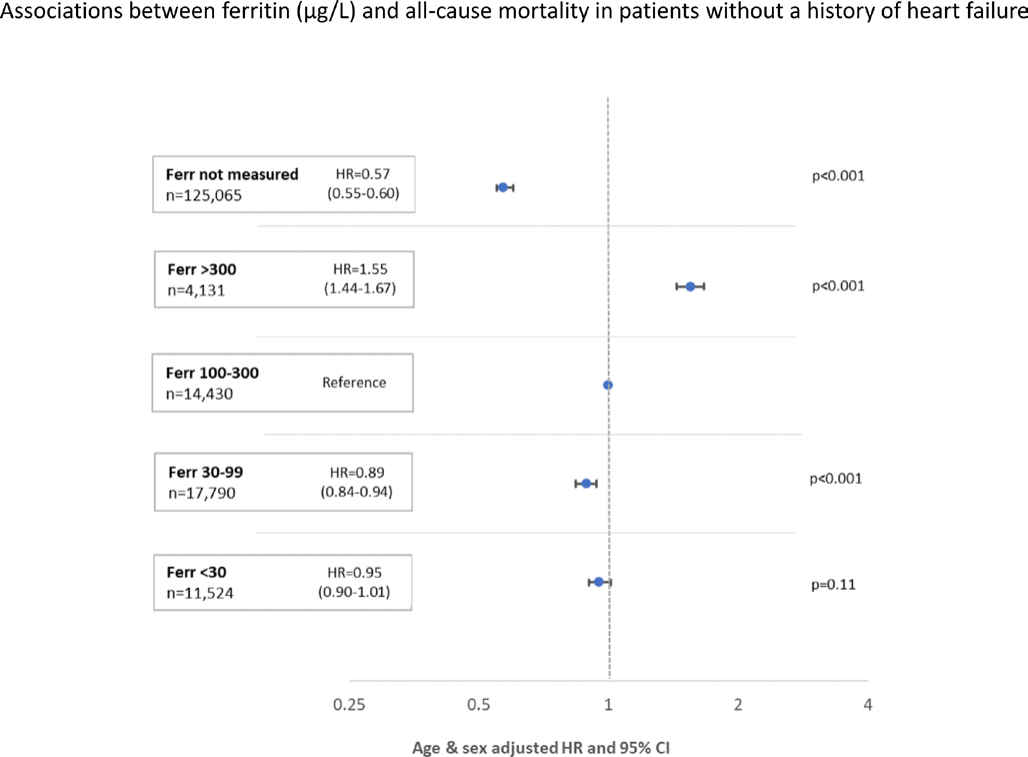
Mortality of patients without a history of heart failure by concentrations of serum ferritin (Ferr). Forest plots showing all-cause mortality from 1 January 2015 to 31 March 2018 for patients without a history of heart failure during, or prior to, 2013/14 according to concentrations of serum Ferr (not measured; >300 µg/L; 100–300 µg/L; 30–100 µg/L; <30 µg/L). Age-adjusted and sex-adjusted HRs with corresponding 95% CIs are presented.

Neither a ferritin <30 µg/L nor a ferritin 30–99 µg/L were associated with higher mortality in any patient group (figure 5 and online supplemental figures S6 and S7). A ferritin >300 µg/L was associated with greater mortality except for those with incident heart failure.

**Figure 6:**
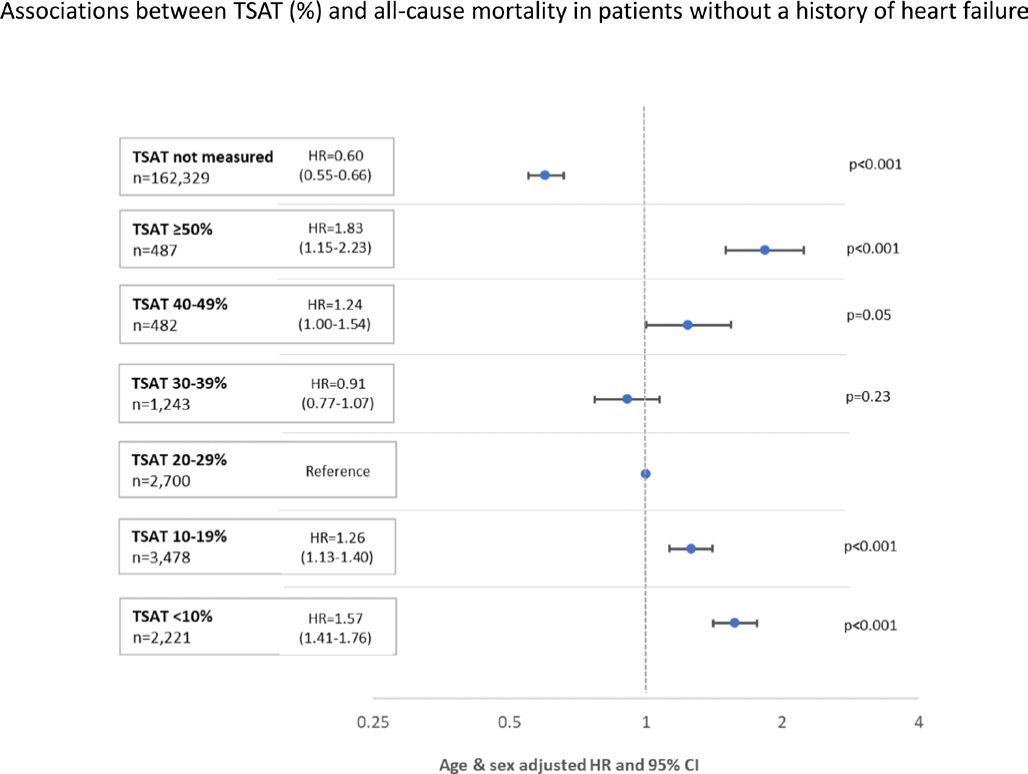
Mortality of patients without a history of heart failure by transferrin saturation (TSAT) (%). Forest plots showing all-cause mortality from 1 January 2015 to 31 March 2018 for patients without a history of heart failure during, or prior to, 2013/14 according to TSAT (%) (not measured; ≥50%; 40%–49%; 30%–39%; 20%–29%; 10%–19%; <10%). Age-adjusted and sex-adjusted HRs with corresponding 95% CIs are presented.

A U-shaped relationship with mortality between both TSAT (figure 6) and serum iron was observed for patients without heart failure, with a nadir of risk at a TSAT between 30% and 39% or a serum iron between 17 and 30 µmol/L (online supplemental figure S8). For patients with heart failure, serum iron and TSAT were rarely measured precluding meaningful analysis (online supplemental figures S6 and S7).

## Discussion

This study has several important findings: (1) many adults with cardiovascular disease living in the West of Scotland have their haemoglobin checked and anaemia is often present, (2) blood tests for iron deficiency are rarely done unless anaemia is severe, (3) anaemia is associated with a higher subsequent incidence of cancer and heart failure, (4) both high and low haemoglobin concentrations are associated with an increased mortality, with the nadir of risk at levels 1–3 g/dL above the WHO criteria for anaemia, (5) a low serum ferritin is associated with a better prognosis but a low TSAT or serum iron with a worse prognosis.

In keeping with previous reports of patients with^8–10^ and without cardiovascular disease,^1 2 11^ lower haemoglobin concentrations were associated with greater mortality in our cohort. However, we found an increase in mortality even when haemoglobin was 0–1 g/dL higher than the current WHO criteria for anaemia. Similar associations between haemoglobin concentrations and mortality have been demonstrated in other large cohorts,^1 11^ and our data add further fuel to the debate^6^ on whether current WHO criteria to diagnose anaemia are appropriate for contemporary clinical practice in older adults. Our findings suggest that the threshold to define anaemia should be raised, at least for adults with cardiovascular disease, by about 1 g/dL for each sex.

In contrast to our findings, some have postulated that the haemoglobin concentration used to define anaemia should be lower than that suggested by the WHO in healthy, younger populations.^12 13^ These studies defined ‘anaemia’ as below the fifth percentiles for age and sex rather than relating haemoglobin concentrations to clinical outcomes. The causes and relative impact of anaemia will vary between healthy individuals and those with chronic cardiovascular disease.

Anaemia in patients with, or at risk of cardiovascular disease is commonly multifactorial in origin and may be a marker of important comorbidity and risk, as well as a therapeutic target.^3^ In our cohort, as haemoglobin decreased, patients were more likely to have diabetes and renal dysfunction, increasing the risk of iron deficiency and defective erythropoiesis.^14^ Rates of GI disease were also higher in those with anaemia. GI disease can cause anaemia in several ways, including malabsorption, increased blood loss and inflammation.^15^ However, patients with anaemia may be more thoroughly investigated than those without anaemia, leading to more diagnoses of GI disease and cancers. Interestingly, we found that a substantial proportion of patients—almost 80% of those with incident heart failure—were prescribed PPIs. PPI prescription rates are increasing in the UK,^16^ often at higher doses and for longer durations than guidelines suggest they should.^17 18^ PPIs reduce gastric acid secretion, which in turn will reduce enteral iron absorption. Although PPIs may reduce GI bleeding in those prescribed antiplatelet or non-steroidal anti-inflammatory drugs, our findings raise concerns that PPI might increase the risk of iron deficiency and anaemia.^17–19^

The incidence of heart failure in our cohort was markedly higher in those with lower haemoglobin concentrations compared with those without anaemia. Low haemoglobin concentration reduces the oxygen carrying capacity of blood. In order to maintain delivery of oxygen to tissues, cardiac output rises, which may increase cardiac work and oxygen demand and may eventually lead to deleterious myocardial remodelling.^3 8^ Iron is also an essential component of the mitochondrial electron transport chain which is responsible for producing 95% of the body’s energy.^20^ These physiological adaptations, coupled with the higher level of comorbidity associated with lower haemoglobin, may help explain our findings.^21^

Although an association between very high haemoglobin concentrations and greater mortality has not been consistently demonstrated,^7 8^ the weight of evidence from multiple epidemiological reports of mostly older patients mirrors our findings.^1 2 9–11^ High haemoglobin concentrations will increase viscosity which may potentiate ischaemic or embolic events^12 13^ or aggravate hypertension.^15^ High haemoglobin concentrations may also be secondary to chronic lung disease or myeloproliferative disorders, which are associated with an increased risk of morbidity and mortality.^14^

This analysis also throws further doubt on the utility of serum ferritin for diagnosing iron deficiency^22^ in patients with cardiovascular disease. Most patients had values <100 µg/L even in the absence of anaemia, many patients with profound anaemia had values >30 µg/L and low values of ferritin were associated with a good prognosis. Serum iron and TSAT were less often measured but were usually low in patients with moderate or severe anaemia, although often low even when haemoglobin was normal. However, unlike serum ferritin, a low serum iron and TSAT were associated with a worse prognosis. Ultimately, bone marrow histology for iron deposits and the clinical response to correction of iron deficiency are the most reliable ways of defining which test should be used to identify iron deficiency.^23^

Iron deficiency—due to medications predisposing to bleeding (antiplatelets) and/or malabsorption of iron (PPIs), reduced dietary intake or the failure to use appropriate body iron stores (eg, due to inflammation)—is a common cause of anaemia in patients with cardiovascular disease.^3 24^ In patients with heart failure, iron deficiency with or without anaemia is associated with worse symptoms, quality of life and greater mortality.^25–27^ Regular testing of iron indices in all patients with heart failure, regardless of their haemoglobin concentration, is suggested by European guidelines.^22^ However, in our cohort, testing for iron deficiency was driven primarily by the severity of anaemia. In contemporary data for patients with heart failure, the rate of testing of iron indices may be as low as 1%–2%^28^ or as high as 27%.^29^ If iron markers are not routinely measured in patients with heart failure, those with iron deficiency may miss out on the benefits of intravenous iron repletion.^30^

### Study limitations

This analysis investigates associations that may or may not be causal on a large population of mostly Caucasian patients with a broad range of cardiovascular conditions. We did not investigate anaemia and iron deficiency in the general population and cannot say whether our findings would differ for people without cardiovascular disease. The lowest value for each test was used to classify patients rather than their average result, which might have led to different findings. Assessment of the relation between some measures of iron deficiency and mortality was limited by low numbers and selective testing. It is possible that some patients were not tested due to frailty or patient choice. We lacked access to records of further diagnostic tests, such as endoscopies, to investigate for co-existent disease. Models were adjusted only for age and sex, as key datasets providing continuous data of potential confounders such as blood pressure or body mass index were not available.

## Conclusion

In patients with a broad range of cardiovascular disorders, including heart failure, haemoglobin is commonly measured but, even when anaemia is profound, iron indices are often not. Haemoglobin concentrations between 0 and 1 g/dL above the WHO definition of anaemia are associated with a worse prognosis, predominantly from cancer and cardiovascular disease, suggesting that the WHO definition of anaemia should be revised, at least for people with cardiovascular disease. A low serum iron concentration or a low TSAT, but not a serum ferritin <100 µg/L, is associated with a worse prognosis.

## Data Availability

All data relevant to the study are included in the article or uploaded as supplementary information.
