# Supplementary material for: Assessment of haemoglobin and serum markers of iron deficiency in people with cardiovascular disease
Source: Heart. 2023 May 2;109(17):1294–301. doi: 10.1136/heartjnl-2022-322145 (PMC10423540; doi:10.1136/heartjnl-2022-322145)
Supplement: Supplementary data [file heartjnl-2022-322145supp001.pdf]

Supplementary Material

The following R packages formed the backbone of the statistical analysis: tidyverse (1), survival (2), RODBC (3), viridis (4), cmprsk (5), lubridate (6), survminer (7), broom (8), reshape2 (9), ggfortify (10), gridExtra (11), forcats (12), and ggpubr (13).

Table S1 Database and codes used to generate the cohort. Patients were included based the existence of a record across at least one of community-based prescriptions and primary care, hospital admission records, and mental health inpatient and day cases care records between 31st December 2009 through 31st March 2018.

Codes are listed based on the formatting used within Safe Haven and unless explicitly noted otherwise, three and four digit ICD-10 codes include all codes below them. BNF codes include all codes below them.

Dataset	Code Type	Variable	Code	Description
SMR01/04	ICD-10	PAD	I73	Other peripheral vascular disease
SMR01/04	ICD-10	CAD	I24	Other Acute ischaemic heart disease
SMR01/04	ICD-10	CAD	I254	Coronary artery aneurism and dissection
SMR01/04	ICD-10	CAD	Z951	Presence of aortocoronary bypass graft
SMR01/04	ICD-10	CAD	Z955	Presence of coronary angioplasty implant and graft
SMR01/04	ICD-10	HF	I50	Heart failure
SMR01/04	ICD-10	HF	I110	Hypertensive heart disease with (congestive) heart failure
SMR01/04	ICD-10	HF	I130	Hypertensive heart and renal disease with (congestive) heart failure
GP LES	Read	PAD	G734	Echocardiogram shows left ventricular systolic dysfunction
GP LES	Read	CAD	G340	Coronary artery disease (CAD)
GP LES	Read	HF	G5yy9	Left ventricular systolic dysfunction (LVSD)
GP LES	Read	HF	585f	Echocardiogram shows left ventricular systolic dysfunction
GP LES	LES Area	HF	4	Heart failure
PIS	BNF	ACEi	0205051	Angiotensin-converting-enzyme inhibitor (ACEi)
PIS	BNF	ARB	0205025	Angiotensin II receptor blocker (ARB)
PIS	BNF	Beta-blocker	0204	Beta-blocker
PIS	BNF	LD	020202	Loop diuretics (LD)
PIS	BNF	MRA	020203	Mineralocorticoid receptor antagonists (MRA)

SMR01, Scottish Morbidity Records for Acute Inpatient and Day Cases; SMR04, Mental Health Inpatient and Day Cases; GP LES, General Practice Local Enhanced Services; PIS, Prescribing Information System for community-based prescriptions; LES Area, local enhanced service area; ICD-10, International Classification of Disease, 10th Version; BNF, British National Formulary; PAD, peripheral arterial disease; CAD, coronary artery disease; HF, heart failure; ACEi, angiotensin-converting-enzyme inhibitor (ACEi); ARB, angiotensin II receptor blocker (ARB); LD, loop diuretic; MRA, mineralocorticoid receptor antagonists.

Table S2: Description of how co-morbidities were defined and the data sources used to define them.

Inclusion reason	Code type	Code	Description
HF	See supplementary table 3		Heart Failure
ARB	See supplementary table 5		Angiotensin II receptor blockers
ACEi	See supplementary table 5		Angiotensin-converting-enzyme inhibitors
LD	See supplementary table 5		Loop diuretics
MRA	See supplementary table 5		Mineralocorticoid-receptor antagonists.
Beta-blocker	See supplementary table 5		Beta blockers
PAD	Read	14F7	H/O: arterial lower limb ulcer
	Read	14NB	H/O: Peripheral vascular disease procedure
	Read	2G63	Ischaemic toe
	Read	7A100	Emerg aortic bypass by anastomosis axillary to femoral art
	Read	7A101	Bypass aorta by anastomosis axillary to femoral artery NEC
	Read	7A102	Axillo-bifemoral bypass graft
	Read	7A103	Axillo-unifemoral PTFE bypass graft
	Read	7A12.00	Other bypass of bifurcation of aorta
	Read	7A120	Emerg bypass bifurc aorta by anast aorta to femoral artery
	Read	7A121	Bypass bifurc aorta by anastom aorta to femoral artery NEC
	Read	7A12111	Aorto bifemoral graft
	Read	7A12112	Dacron aortofemoral Y graft
	Read	7A123	Bypass bifurcation aorta by anastom aorta to iliac artery
	Read	7A12311	Aorto biiliac graft
	Read	7A12312	Dacron aortoiliac Y graft
	Read	7A12y	Other specified other bypass of bifurcation of aorta
	Read	7A12z	Other bypass of bifurcation of aorta NOS
	Read	7A192	Open embolectomy of bifurcation of aorta
	Read	7A41	Other bypass of iliac artery
	Read	7A410	Emerg bypass iliac art by iliac/femoral art anastomosis NEC
	Read	7A411	Bypass iliac artery by iliac/femoral artery anastomosis NEC
	Read	7A412	Emerg bypass iliac artery by femoral/femoral art anast NEC
	Read	7A41211	Emergency femoro-femoral prosthetic cross over graft
	Read	7A413	Bypass iliac artery by femoral/femoral art anastomosis NEC
	Read	7A41311	Femoro-femoral prosthetic cross over graft
	Read	7A414	Emerg bypass comm iliac art by aorta/com iliac art anast NEC
	Read	7A416	Emerg bypass leg artery by aorta/com fem art anastomosis NEC
	Read	7A419	Bypass common iliac artery by aorta/com iliac art anast NEC
	Read	7A41B	Bypass leg artery by aorta/com femoral art anastomosis NEC
	Read	7A41C	Bypass leg artery by aorta/deep femoral art anastomosis NEC
	Read	7A41F	Ilio-femoral prosthetic cross over graft
	Read	7A41y	Other specified other bypass of iliac artery
	Read	7A41z	Other bypass of iliac artery NOS
	Read	7A42	Reconstruction of iliac artery
	Read	7A420	Endarterectomy and patch repair of iliac artery
	Read	7A42011	Endarterectomy and patch repair of common iliac artery

PAD	Read	7A42012	Iliac endarterectomy and patch
	Read	7A421	Endarterectomy of iliac artery NEC
	Read	7A42111	Endarterectomy of common iliac artery NEC
	Read	7A42y	Other specified reconstruction of iliac artery
	Read	7A42z	Reconstruction of iliac artery NOS
	Read	7A43	Other open operations on iliac artery
	Read	7A430	Repair of iliac artery NEC
	Read	7A43011	Repair of common iliac artery NEC
	Read	7A431	Open embolectomy of iliac artery
	Read	7A43111	Open embolectomy of common iliac artery
	Read	7A433	Open insertion of iliac artery stent
	Read	7A440	Percutaneous transluminal angioplasty of iliac artery
	Read	7A441	Percutaneous transluminal embolectomy of iliac artery
	Read	7A443	Insertion of iliac artery stent
	Read	7A444	Percutaneous transluminal insertion of iliac artery stent
	Read	7A44y	Other specified transluminal operation on iliac artery
	Read	7A44z	Transluminal operation on iliac artery NOS
	Read	7A47	Other emergency bypass of femoral artery or popliteal artery
	Read	7A470	Emerg bypass femoral art by fem/pop art anast c prosth NEC
	Read	7A471	Emerg bypass popliteal art by pop/pop art anast c prosth NEC
	Read	7A472	Emerg bypass femoral art by fem/pop a anast c vein graft NEC
	Read	7A473	Emerg bypass pop art by pop/pop art anast c vein graft NEC
	Read	7A474	Emerg bypass femoral art by fem/tib art anast c prosth NEC
	Read	7A476	Emerg bypass femoral art by fem/tib a anast c vein graft NEC
	Read	7A477	Emerg bypass pop art by pop/tib art anast c vein graft NEC
	Read	7A47B	Emerg bypass pop art by pop/peron art anast c vein graft NEC
	Read	7A47C	Emerg bypass femoral artery by fem/fem art anastomosis NEC
	Read	7A47D	Emerg bypass popliteal artery by pop/fem art anastomosis NEC
	Read	7A47y	Other emergency bypass of femoral or popliteal artery OS
	Read	7A47z	Other emergency bypass of femoral or popliteal artery NOS
	Read	7A48	Other bypass of femoral artery or popliteal artery
	Read	7A480	Bypass femoral artery by fem/pop art anast c prosthesis NEC
	Read	7A481	Bypass popliteal artery by pop/pop a anast c prosthesis NEC
	Read	7A482	Bypass femoral artery by fem/pop art anast c vein graft NEC
	Read	7A483	Bypass popliteal artery by pop/pop a anast c vein graft NEC
	Read	7A484	Bypass femoral artery by fem/tib art anast c prosthesis NEC
	Read	7A485	Bypass popliteal artery by pop/tib a anast c prosthesis NEC
	Read	7A486	Bypass femoral artery by fem/tib art anast c vein graft NEC
	Read	7A487	Bypass popliteal artery by pop/tib a anast c vein graft NEC
	Read	7A488	Bypass femoral artery by fem/peron a anast c prosthesis NEC
	Read	7A48A	Bypass femoral artery by fem/peron a anast c vein graft NEC
	Read	7A48B	Bypass popliteal art by pop/peron art anast c vein graft NEC
	Read	7A48C	Bypass femoral artery by femoral/femoral art anastomosis NEC
	Read	7A48D	Bypass popliteal artery by pop/fem artery anastomosis NEC
	Read	7A48E	Femoro-femoral prosthetic cross over graft
	Read	7A48y	Other bypass of femoral artery or popliteal artery OS

	Read	7A48z	Other bypass of femoral artery or popliteal artery NOS
	Read	7A49	Reconstruction of femoral artery or popliteal artery
	Read	7A490	Endarterectomy and patch repair of femoral artery
	Read	7A491	Endarterectomy and patch repair of popliteal artery
	Read	7A492	Endarterectomy of femoral artery NEC
	Read	7A493	Endarterectomy of popliteal artery NEC
	Read	7A494	Profundoplasty femoral artery & patch repair deep fem artery
	Read	7A495	Profundoplasty and patch repair of popliteal artery
	Read	7A496	Profundoplasty of femoral artery NEC
	Read	7A497	Profundoplasty of popliteal artery NEC
	Read	7A498	Reconstruction of femoral artery with vein graft
	Read	7A499	Reconstruction of popliteal artery with vein graft
	Read	7A49y	Reconstruction of femoral or popliteal artery OS
	Read	7A49z	Reconstruction of femoral or popliteal artery NOS
	Read	7A4A	Other open operations on femoral artery or popliteal artery
	Read	7A4A0	Repair of femoral artery NEC
	Read	7A4A1	Repair of popliteal artery NEC
	Read	7A4A2	Open embolectomy of femoral artery
	Read	7A4A211	Open thrombectomy of femoral artery
	Read	7A4A212	Open femoral embolectomy
	Read	7A4A3	Open embolectomy popliteal artery
	Read	7A4A311	Open thrombectomy of popliteal artery
	Read	7A4A7	Repair of femoral artery with temporary silastic shunt
PAD	Read	7A4A8	Repair of popliteal artery with temporary silastic shunt
	Read	7A4Ay	Other open operation on femoral or popliteal artery OS
	Read	7A4B0	Percutaneous transluminal angioplasty of femoral artery
	Read	7A4B1	Percutaneous transluminal angioplasty of popliteal artery
	Read	7A4B2	Percutaneous transluminal embolectomy of femoral artery
	Read	7A4B3	Percutaneous transluminal embolectomy of popliteal artery
	Read	7A4B4	Percutaneous transluminal embolisation of femoral artery
	Read	7A4B5	Percutaneous transluminal embolisation of popliteal artery
	Read	7A4B8	Percut translum thrombolysis femoral graft streptokinase
	Read	7A4B9	Percutaneous transluminal insertion of stent femoral artery
	Read	7A50	Revision of reconstruction of artery
	Read	7A500	Revision of reconstruction involving aorta
	Read	7A501	Revision of reconstruction involving iliac artery
	Read	7A502	Revision of reconstruction involving femoral artery
	Read	7A503	Revision of reconstruction of popliteal artery
	Read	A3A0F	Gas gangrene-foot
	Read	C107	Diabetes mellitus with peripheral circulatory disorder
	Read	C1070	Diabetes mellitus, juvenile +peripheral circulatory disorder
	Read	C1071	Diabetes mellitus, adult, + peripheral circulatory disorder
	Read	C1073	IDDM with peripheral circulatory disorder
	Read	C1074	NIDDM with peripheral circulatory disorder
	Read	C107z	Diabetes mellitus NOS with peripheral circulatory disorder
	Read	C108G	Insulin dependent diab mell with peripheral angiopathy
PAD			

	Read	C109F	Non-insulin-dependent d m with peripheral angiopath
	Read	C109F11	Type II diabetes mellitus with peripheral angiopathy
	Read	C109F12	Type 2 diabetes mellitus with peripheral angiopathy
	Read	C10EG	Type 1 diabetes mellitus with peripheral angiopathy
	Read	C10FF	Type 2 diabetes mellitus with peripheral angiopathy
	Read	G700	Aorto-iliac disease
	Read	G702	Extremity artery atheroma
	Read	G702z	Extremity artery atheroma NOS
	Read	G73	Other peripheral vascular disease
	Read	G731	Thromboangiitis obliterans
	Read	G7310	Buerger's disease
	Read	G731z	Thromboangiitis obliterans NOS
	Read	G732	Peripheral gangrene
	Read	G7320	Gangrene of toe
	Read	G7321	Gangrene of foot
	Read	G733	Ischaemic foot
	Read	G73y	Other specified peripheral vascular disease
	Read	G73y0	Diabetic peripheral angiopathy
	Read	G73y1	Peripheral angiopathic disease EC NOS
	Read	G73y2	Acrocyanosis
	Read	G73y4	Acroparaesthesia - Schultze's type
	Read	G73y5	Acroparaesthesia - Nothnagel's type
	Read	G73y511	Nothnagel's vasomotor acroparaesthesia
	Read	G73y6	Acroparaesthesia - unspecified
	Read	G73y7	Erythrocyanosis
	Read	G73y8	Erythromelalgia
	Read	G73yz	Other specified peripheral vascular disease NOS
	Read	G73z	Peripheral vascular disease NOS
	Read	G73z0	Intermittent claudication
	Read	G73z011	Claudication
	Read	G73zz	Peripheral vascular disease NOS
	Read	G740	Aortoiliac obstruction
	Read	G7424	Embolism and thrombosis of the femoral artery
	Read	G7425	Embolism and thrombosis of the popliteal artery
	Read	G7426	Embolism and thrombosis of the anterior tibial artery
	Read	G7427	Embolism and thrombosis of the dorsalis pedis artery
	Read	G7429	Embolism and thrombosis of a leg artery NOS
	Read	G742z	Peripheral arterial embolism and thrombosis NOS
	Read	G74y0	Embolism and/or thrombosis of the common iliac artery
	Read	G74y1	Embolism and/or thrombosis of the internal iliac artery
	Read	G74y2	Embolism and/or thrombosis of the external iliac artery
	Read	G74y3	Embolism and thrombosis of the iliac artery unspecified
	Read	Gyu74	[X]Other specified peripheral vascular diseases
	Read	M271	Ischaemic leg ulcer
PAD	Read	M2710	Ischaemic ulcer diabetic foot
	Read	M2713	Arterial leg ulcer

	Read	M2714	Mixed venous and arterial leg ulcer
	Read	R0542	[D]Gangrene of toe in diabetic
	Read	R0543	[D]Widespread diabetic foot gangrene
	Read	R0550	[D]Failure of peripheral circulation
	Read	R055011	[D]Peripheral circulatory failure
	ICD-10	I731	Thromboangiitis obliterans [Buerger]
	ICD-10	I738	Other specified peripheral vascular diseases
	ICD-10	I739	Peripheral vascular disease, unspecified
	ICD-10	I743	Embolism and thrombosis of arteries of lower extremities
	ICD-10	I744	Embolism and thrombosis of arteries of extremities, unspecified
	ICD-10	I745	Embolism and thrombosis of iliac artery
	OPCS-4	L50	Other emergency bypass of iliac artery
	OPCS-4	L51	Other bypass of iliac artery
	OPCS-4	L52	Reconstruction of iliac artery
	OPCS-4	L530	Other open operations on iliac artery
	OPCS-4	L531	Repair of iliac artery NEC
	OPCS-4	L532	Open embolectomy of iliac artery
	OPCS-4	L541	Percutaneous transluminal angioplasty of iliac artery
	OPCS-4	L542	Percutaneous transluminal embolectomy of iliac artery
	OPCS-4	L544	Percutaneous transluminal insertion of stent into iliac artery
	OPCS-4	L548	Other specified transluminal operations on iliac artery
	OPCS-4	L549	Unspecified transluminal operations on iliac artery
	OPCS-4	L58	Other emergency bypass of femoral artery
	OPCS-4	L59	Other bypass of femoral artery
	OPCS-4	L60	Reconstruction of femoral artery
	OPCS-4	L620	Other open operations on femoral artery
	OPCS-4	L621	Repair of femoral artery NEC
	OPCS-4	L622	Open embolectomy of femoral artery
	OPCS-4	L628	Other specified other open operations on femoral artery
	OPCS-4	L629	Unspecified other open operations on femoral artery
	OPCS-4	L631	Percutaneous transluminal angioplasty of femoral artery
	OPCS-4	L632	Percutaneous transluminal embolectomy of femoral artery
	OPCS-4	L633	Percutaneous transluminal embolisation of femoral artery
	OPCS-4	L635	Percutaneous transluminal insertion of stent into femoral artery
	OPCS-4	L650	Revision of reconstruction of artery
	OPCS-4	L651	Revision of reconstruction involving aorta
	OPCS-4	L652	Revision of reconstruction involving iliac artery
	OPCS-4	L653	Revision of reconstruction involving femoral artery
CAD	ICD-10	I20	Angina pectoris
	ICD-10	I21	Acute myocardial infarction
	ICD-10	I22	Subsequent myocardial infarction
	ICD-10	I23	Certain current complications following acute myocardial infarction
	ICD-10	I24	Other acute ischaemic heart diseases
	ICD-10	I25	Chronic ischaemic heart disease
CAD	Read	14AL	H/O: Treatment for ischaemic heart disease
	Read	G310	Dressler's syndrome
	Read	G33z5	Post infarct angina
	Read	889A	Diab mellit insulin-glucose infus acute myocardial infarct

	Read	6A2	Coronary heart disease annual review
	Read	6A4	Coronary heart disease review
	Read	8B3k	Coronary heart disease medication review
	Read	8H2V	Admit ischaemic heart disease emergency
	Read	G3	Ischaemic heart disease
	Read	G31	Other acute and subacute ischaemic heart disease
	Read	G3110	Myocardial infarction aborted
	Read	G311011	MI - myocardial infarction aborted
	Read	G31y	Other acute and subacute ischaemic heart disease
	Read	G31y2	Subendocardial ischaemia
	Read	G31y3	Transient myocardial ischaemia
	Read	G31yz	Other acute and subacute ischaemic heart disease NOS
	Read	G34	Other chronic ischaemic heart disease
	Read	G340	Coronary atherosclerosis
	Read	G344	Silent myocardial ischaemia
	Read	G34y	Other specified chronic ischaemic heart disease
	Read	G34y1	Chronic myocardial ischaemia
	Read	G34yz	Other specified chronic ischaemic heart disease NOS
	Read	G34z	Other chronic ischaemic heart disease NOS
	Read	G34z0	Asymptomatic coronary heart disease
	Read	G3y	Other specified ischaemic heart disease
	Read	G3z	Ischaemic heart disease NOS
	Read	Gyu3	[X]Ischaemic heart diseases
	Read	Gyu32	[X]Other forms of acute ischaemic heart disease
	Read	Gyu33	[X]Other forms of chronic ischaemic heart disease
	Read	G300	Acute anterolateral infarction
	Read	G301	Other specified anterior MI
	Read	G3010	Acute anteroapical infarction
	Read	G3011	Acute anteroseptal infarction
	Read	G301z	Anterior MI NOS
	Read	G302	Acute inferolateral infarction
	Read	G303	Acute inferoposterior infarction
	Read	G304	Posterior MI NOS
	Read	G305	Lateral MI NOS
	Read	G306	True posterior MI
	Read	G307	Acute subendocardial infarction
	Read	G3070	Acute non-Q wave infarction
	Read	G3071	Acute non-ST segment elevation MI
	Read	G308	Inferior MI NOS
	Read	G309	Acute Q-wave infarct
	Read	G30A	Mural thrombosis
CAD	Read	G30B	Acute posterolateral MI
	Read	G30X	Acute transmural MI of unspecif site
	Read	G30X0	Acute ST segment elevation MI
	Read	G30y	Other acute MI
	Read	G30z	Acute MI NOS

Read	G311	Unstable angina
Read	G3112	Angina at rest
Read	G3113	Refractory angina
Read	G3114	Worsening angina
Read	G3115	Acute coronary syndrome
Read	G31y0	Acute coronary syndrome
Read	G33	Angina pectoris
Read	G33z	Angina pectoris NOS
Read	G33z3	Angina on effort
Read	G33z4	Ischaemic chest pain
Read	G33zz	Angina pectoris NOS
Read	G3400	Single coronary vessel disease
Read	G3401	Double coronary vessel disease
Read	G70y011	Carotid artery disease
Read	G30	Acute MI
Read	G32	Old MI
Read	G32	Old MI

ICD-10, International Classification of Disease, 10th Version; BNF, British National Formulary; PAD, peripheral arterial disease; CAD, coronary artery disease; HF, heart failure; ACEi, angiotensin-converting-enzyme inhibitor (ACEi); ARB, angiotensin II receptor blocker (ARB); LD, loop diuretic; MRA, mineralocorticoid receptor antagonists.

Table S3: ICD-10 and Greater Glasgow and Clyde NHS formatted Read Codes used to identify patients with heart failure.

Code Type	Code	Description	Conrad et al	Included
ICD-10	I50	Heart Failure	1	1
ICD-10	I420	Dilated cardiomyopathy (congestive cardiomyopathy	1	1
ICD-10	I429	Cardiomyopathy, unspecified (Cardiomyopathy (primary)(secondary) NOS)	1	1
ICD-10	I110	Hypertensive heart disease with (congestive) heart failure	1	1
ICD-10	I255	Ischaemic cardiomyopathy	1	1
ICD-10	I132	Hypertensive heart and renal disease with both (congestive) heart failure and renal failure	1	1
ICD-10	I130	Hypertensive heart and renal disease with (congestive) heart failure	1	1
Read	585f	Echocardiogram shows left ventricular systolic dysfunction	1	1
Read	G58	Heart Failure	1	1
Read	G581	Left ventricular failure	1	1
Read	662g	NYHA classification class II	1	1
Read	662f	NYHA classification - class I	1	1
Read	G5yy9	Left ventricular systolic dysfunction	1	1
Read	G580	Congestive heart failure	1	1
Read	662h	NYHA classification- class III	1	1
Read	9hH1	Excepted heart failure quality indicators: informed dissent	1	1
Read	9hH0	Excepted heart failure quality indicators: patient unsuitable	1	1
Read	21264	Heart Failure resolved	1	1
Read	G5802	Decompensated cardiac failure	1	1
Read	G58z	Heart failure NOS	1	1
Read	662i	NYHA classification - class IV	1	1
Read	G5801	Chronic congestive heart failure	1	1
Read	8HHb	Referral to heart failure nurse	1	1
Read	G5810	Acute left ventricular failure	1	1
Read	G343	Ischaemic cardiomyopathy	1	1
Read	G582	Acute heart failure	1	1
Read	G5800	Acute congestive heart failure	0	1
Read	G583	Heart failure with normal ejection fraction	1	1
Read	585g	Echocardiogram shows left ventricular diastolic dysfunction	1	1
Read	G584	Right ventricular failure	1	1
Read	G5yyA	Left ventricular diastolic dysfunction	1	1
Read	G5544	Primary dilated cardiomyopathy	1	1
Read	G55z	Cardiomyopathy NOS	1	1
Read	G5803	Compensated cardiac failure	1	1
Read	G5804	Congestive heart failure due to valvular disease	1	1
Read	G555	Alcoholic cardiomyopathy	1	1
Read	G551	Hypertrophic obstructive cardiomyopathy	1	1
Read	G5yyD	Left ventricular cardiac dysfunction	1	1
Read	9hH	Exception reporting heart failure quality indicators	1	1
Read	G1yz1	Pneumatic left ventricular failure	1	1
Read	679X	Heart failure education	1	1

Read	8HBE	Heart failure follow-up	1	1
Read	1O1	Heart failure confirmed	1	1
Read	8H2S	Admit heart failure emergency	1	1
Read	8CMK	Has heart failure management plan	1	1
Read	9Or5	Heart failure monitoring third letter	1	1
Read	9Or3	Heart failure monitoring first letter	1	1
Read	9Or4	Heart failure monitoring second letter	1	1
Read	9Or2	Heart failure monitoring verbal invite	1	1
Read	9Or	Heart failure monitoring administration	1	1
Read	9Or1	Heart failure monitoring telephone invite	1	1
Read	8HgD	Discharge from heart failure nurse service	1	1
Read	8HHz	Referral to heart failure exercise programme	1	1
Read	8CL3	Heart failure care plan discussed with patient	1	1
Read	8IE1	Referral to heart failure exercise program declined	1	1
Read	8IB8	Referral to heart failure exercise program not indicated	1	1
Read	8CeC	Preferred place of care for next exacerbation heart failure	1	1
Read	G55	Cardiomyopathy	1	1
Read	G5540	Congestive Cardiomyopathy	1	1
Read	G559	Arrhythmogenic right ventricular cardiomyopathy	1	1
Read	33BA	Impaired left ventricular function	1	1
Read	G41z	Chronic cor pulmonale	1	1
Read	G5543	Hypertrophic non-obstructive cardiomyopathy	1	1
Read	G55y	Secondary cardiomyopathy NOS	1	1
Read	G5542	Familial cardiomyopathy	1	1
Read	8CMW8	Heart failure clinical pathway	1	1
Read	G5581	Cardiomyopathy in myotonic dystrophy	1	1
Read	Gyu5M	[X] Other hypertrophic cardiomyopathy	1	1
Read	G2111	Benign hypertensive heart disease with CCF	1	1
Read	G21z1	Hypertensive heart disease NOS with CCF	1	1
Read	G232	hypertensive heart and renal disease with (congestive) heart failure	1	1
Read	662T	Congestive heart failure monitoring	1	1
Read	662W	Heart failure annual review	1	1
Read	662p	Heart failure 6 month review	1	1
Read	9On	Left ventricular dysfunction monitoring administration	1	1
Read	9On0	Left ventricular dysfunction monitoring 1st letter	1	1
Read	9On1	Left ventricular dysfunction monitoring 2nd letter	1	1
Read	9On2	Left ventricular dysfunction monitoring 3rd letter	1	1
Read	9On3	Left ventricular dysfunction monitoring verbal invite	1	1
Read	9On4	Left ventricular dysfunction monitoring telephone invite	1	1
Read	9N4s	Did not attend practice nurse heart failure clinic	1	1
Read	8B29	Cardiac failure therapy	1	1
Read	23E1	O/E -pulmonary oedema	1	1
Read	9h1	Exception reporting: LVD quality indicators	1	1
Read	9h12	Excepted from LVD quality indicators: informed dissent	1	1
Read	9h11	Excepted from LVD quality indicators: Patient unsuitable	1	1

Read	14A6	H/O: heart failure	1	1
Read	14AM	H/O: heart failure in last year	1	1
Read	8HTL0	Referral to rapid access heart failure clinic	1	1
Read	8IE0	Referral to heart failure education group declined	1	1
Read	G234	Hyperten heart & renal dis + both (congestv) heart and renal fail	0	1
Read	12CR	HF: Hypertrophic obstructive cardiomyopathy	1	1

Table S4: Greater Glasgow and Clyde formatted Read Codes used to exclude heart failure cases where the code refers to an existing diagnosis after 31st December 2012.

Code	Description	Conrad et al	Included
9N4s	Did not attend practice nurse heart failure clinic	1	1
8B29	Cardiac failure therapy	1	1
679X	Heart failure education	1	1
8HHb	Referral to heart failure nurse	1	1
23E1	O/E – pulmonary oedema	1	1
9hH	Exception reporting: heart failure quality indicators	1	1
9hH0	Excepted heart failure quality indicators: Patient unsuitable	1	1
9h1	Exception reporting: LVD quality indicators	1	1
9h11	Exception from LVD quality indicators: Patient unsuitable	1	1
9h12	Exception from LVD quality indicators: Informed dissent	1	1
14A6	H/O: heart failure	1	1
14AM	H/O: Heart failure in last year	1	1
662p	Heart failure 6 month review	1	1
662T	Congestive heart failure monitoring	1	1
662W	Heart failure annual review	1	1
8CL3	Heart failure care plan discussed with patient	1	1
8HBE	Heart failure follow-up	1	1
8HHz	Referral to heart failure exercise programme	1	1
8Hk0	Referred to heart failure education group	1	1
9hH1	Excepted heart failure quality indicators: Informed dissent	1	1
9Or	Heart failure monitoring administration	1	1
9Or0	Heart failure review completed	1	1
9Or1	Heart failure monitoring telephone invite	1	1
9Or2	Heart failure monitoring verbal invite	1	1
9Or3	Heart failure monitoring first letter	1	1
9Or4	Heart failure monitoring second letter	1	1
9Or5	Heart failure monitoring third letter	1	1
9On	Left ventricular dysfunction monitoring administration	1	1
9On0	Left ventricular dysfunction monitoring first letter	1	1
9On1	Left ventricular dysfunction monitoring second letter	1	1
9On2	Left ventricular dysfunction monitoring third letter	1	1
9On3	Left ventricular dysfunction monitoring verbal invite	1	1
9On4	Left ventricular dysfunction monitoring telephone invite	1	1
2126400	Heart Failure Resolved	1	1
21264	Heart Failure Resolved	1	1
8HgD	Discharged from heart failure nurse service	1	1
8HTL000	Referral to rapid access heart failure clinic	1	1
8HTL0	Referral to rapid access heart failure clinic	1	1
8IB8	Referral to heart failure exercise programme not indicated	1	1
8IE1	Referral to heart failure excursive programme declined	1	1
81E0	Referral to heart failure education group declined	1	1
12CR	FH: Hypertrophic obstructive cardiomyopathy	1	1

Table S5: Prescribed medication classifications, categories each medication was assigned to, the portion of the BNF code used to select for said category, and the description of the BNF selection code.

Category	BNF selection code	Description
ACEi	0205051	Angiotensin converting enzyme
Antiplatelet	0209	Antiplatelet
ARB	0205052<br>0206020Z0	Angiotensin receptor blockers<br>Valsartan/Amlodipine
Beta-blocker	0204	Beta-adrenergic blocking agents
Bronchodilators	0301	Bronchodilators
B12	0901020D0<br>0901020N0	Cyanocobalamin<br>Hydroxocobalamin
Calcium-channel blockers	020602<br>0205052AB<br>0205052AC	Calcium-channel blockers<br>Olmesartan medoxomil/amlodipine<br>Olmesartan medoxomil/amlodipine/hydrochlorothiazide
	0206020C0<br>0206020T0	Diltiazem hydrochloride<br>Verapamil hydrochloride
Digoxin	0201010F	Digoxin
Hypoglycemics	060101	Insulin
	060102	Other hypoglycaemics
Folate	0901020G0	Folic Acid
Lipid-regulating	0212	Lipid-regulating
Loop diuretics	020202<br>0202040D0<br>0202040B0<br>0202040T0<br>0202040U0<br>0202080D0<br>0202080C0<br>0202080K0	Loop diuretics<br>Amiloride HCI with loop diuretics<br>Co-amilofruse (Amiloride hydrochloride/furosemide)<br>Spironolactone with loop diuretics<br>Triamterene with loop diuretics<br>Bumetanide/Amiloride hydrochloride<br>Bumetanide/potassium<br>Furosemide/potassium
Low dose aspirin	0202000A0<br>0204000AC<br>0209000V0	Aspirin<br>Bisoprolol fumarate/aspirin<br>Dipyridamole and Aspirin
MRA	0202030X0<br>0202030S0<br>0202040G0<br>0202040T0<br>0202040S0	Eplerenone<br>Spironolactone<br>Co-flumactone(Hydroflumethiazide/spironolactone)<br>Spironolactone with loop diuretics<br>Spironolactone with thiazides
NSAIDs	100101	NSAIDs
Oral anticoagulants	020802	Oral anticoagulants
Oral Iron	0901011C0<br>0901011Y0<br>0901011X0<br>0901011F0<br>0901011H0<br>0901011K0<br>0901011P0	Compound iron prescriptions<br>Ferric maltol<br>Ferrous calcium citrate<br>Ferrous fumarate<br>Ferrous gluconate<br>Ferrous glycine sulphate<br>Ferrous sulphate
PPI	010305	Proton pump inhibitors
H2 antagonists	010301	H2-Receptro Antagonists

BNF, British National Formulary; ACEi, Angiotensin-converting enzyme inhibitors; ARB, Angiotensin II receptor blocker; MRA, Mineralocorticoid receptor antagonists; NSAIDs, non-steroidal anti inflammatory drugs

Table S6: Characteristics of those without heart failure – Survivors at 31/12/2014 (all data listed on or before 31/12/2014 unless stated otherwise)								
WHO anaemia Class (Nadir 2013 to 2014)	No Hb measured	>4 above	3-4 above	1-3 above	0-1 above	0-1 below	1-2 below	>2 below
N=	40,740 (24%)	1,374 (1%)	6,325 (4%)	55,166 (32%)	34,025 (20%)	17,462 (10%)	9,308 (5%)	8,540 (5%)
Age (years) in 2013	62 (56-70)	62 (55-69)	62 (56-69)	64 (57-72)	67 (59-76)	71 (62-79)	73 (64-80)	73 (65-81)
Sex (women)	20,606 (51%)	448 (33%)	2,498 (39%)	29,812 (54%)	20,848 (61%)	10,927 (63%)	5,592 (60%)	4,549 (53%)
Diabetes or Hypoglycaemic Therapy	4,671 (11%)	239 (17%)	1,090 (17%)	9,847 (18%)	7,019 (21%)	4,313 (25%)	2,569 (28%)	2,411 (28%)
IHD	4,741 (12%)	230 (17%)	1,108 (18%)	11,232 (20%)	8,589 (25%)	4,973 (28%)	2,861 (31%)	2,692 (32%)
COPD	2,430 (6%)	214 (16%)	818 (13%)	6,524 (12%)	4,729 (14%)	2,927 (17%)	1,767 (19%)	1,765 (21%)
eGFR (last available)	82 (72-93)	82 (71-94)	82 (72-93)	81 (70-92)	78 (67-90)	74 (62-87)	72 (57-87)	73 (58-88)
GI disease	679 (2%)	38 (3%)	158 (2%)	1,609 (3%)	1,370 (4%)	923 (5%)	700 (8%)	1,071 (13%)
Any cancer prior to 2013	1,472 (4%)	58 (4%)	246 (4%)	2,960 (5%)	2,377 (7%)	1,555 (9%)	1,011 (11%)	1,101 (13%)
Any incident cancer 2013/14	49 (0%)	11 (1%)	44 (1%)	577 (1%)	630 (2%)	618 (4%)	537 (6%)	910 (11%)
**ECG (last result prior to 1st January 2015)**								
AF/Flutter	222/3,271 (7%)	45/361 (12%)	138/1,536 (9%)	1,118/15,526 (7%)	887/11,776 (8%)	604/7,276 (8%)	410/4,413 (9%)	466/4,863 (10%)
**Haemoglobin prior to 1st January 2013**								
Hb 4g/dL above WHO	296 (1%)	271 (26%)	266 (5%)	197 (0%)	35 (0%)	18 (0%)	<6 (NA%)	13 (0%)
Hb 3-4g/dL above WHO	1,439 (6%)	360 (35%)	1,401 (29%)	1,813 (4%)	162 (1%)	96 (1%)	30 (0%)	31 (0%)
Hb 1-3g/dL above WHO	12,165 (52%)	324 (31%)	2,794 (57%)	29,661 (65%)	7,586 (25%)	1,866 (12%)	810 (9%)	718 (9%)
Hb 0-1g/dL above WHO	5,835 (25%)	28 (3%)	250 (5%)	10,049 (22%)	13,954 (47%)	4,799 (30%)	1,537 (18%)	1,168 (15%)
Anaemia prior to 2013 (% of those tested)	3,726 (16%)	53 (5%)	203 (4%)	4,243 (9%)	8,265 (28%)	9,226 (58%)	6,238 (72%)	5,850 (75%)
**Haematinic profile (Between 2013 and 2014)**								
Ferritin (median (Q1-Q3))	107 (62-197)	158 (86-291)	128 (75-217)	96 (52-171)	74 (38-141)	61 (26-133)	47 (18-123)	35 (12-120)
Iron (median (Q1-Q3))	16 (12-20)	18 (15-23)	19 (14-24)	16 (12-20)	14 (10-18)	11 (8-16)	9 (6-13)	7 (4-12)
Transferrin (median (Q1-Q3))	2.4 (2.1-2.7)	2.3 (2.1-2.6)	2.5 (2.1-2.7)	2.4 (2.2-2.7)	2.4 (2.0-2.7)	2.3 (2.0-2.7)	2.3 (2.0-2.8)	2.3 (1.8-2.8)
TSAT (median (Q1-Q3))	28 (22-34)	29 (26-37)	30 (22-38)	26 (20-33)	22 (15-31)	19 (12-27)	16 (10-22)	12 (6-21)
B12 (median (Q1-Q3))	313 (234-433)	335 (249-446)	350 (265-464)	344 (259-456)	336 (252-461)	328 (244-447)	320 (238-438)	316 (230-449)
Folate (median (Q1-Q3))	5.7 (4.0-7.8)	3.9 (2.7-6.0)	4.4 (3.1-6.7)	4.9 (3.3-7.4)	5.0 (3.5-7.8)	4.9 (3.3-7.4)	4.7 (3.2-7.5)	4.4 (2.9-7.0)

Serum sodium	139 (137-140)	138 (137-140)	138 (137-140)	138 (137-140)	138 (136-139)	137 (134-139)	136 (133-138)	135 (132-137)
Albumin	38 (36-40)	38 (36-40)	38 (36-40)	37 (36-39)	36 (34-38)	35 (33-37)	34 (31-36)	31 (27-35)
**Prescriptions (on 1st January 2015)**								
Iron (oral)	149 (0%)	<6 (NA%)	26 (0%)	493 (1%)	887 (3%)	1,314 (8%)	1,717 (18%)	2,901 (34%)
B12	259 (1%)	18 (1%)	72 (1%)	941 (2%)	1,054 (3%)	825 (5%)	632 (7%)	704 (8%)
Folate	272 (1%)	59 (4%)	189 (3%)	1,807 (3%)	1,889 (6%)	1,663 (10%)	1,370 (15%)	1,690 (20%)
Loop diuretics	1,632 (4%)	87 (6%)	382 (6%)	4,197 (8%)	3,843 (11%)	2,593 (15%)	1,775 (19%)	1,911 (22%)
ACEi/ARB	18,629 (46%)	681 (50%)	3,285 (52%)	28,782 (52%)	18,284 (54%)	9,578 (55%)	5,076 (55%)	4,242 (50%)
BB	11,367 (28%)	412 (30%)	1,991 (31%)	18,206 (33%)	12,061 (35%)	6,092 (35%)	3,265 (35%)	2,962 (35%)
MRA	115 (0%)	14 (1%)	35 (1%)	337 (1%)	311 (1%)	188 (1%)	142 (2%)	248 (3%)
Antiplatelets	8,303 (20%)	417 (30%)	1,907 (30%)	18,245 (33%)	13,503 (40%)	7,892 (45%)	4,439 (48%)	3,986 (47%)
OAC	890 (2%)	95 (7%)	312 (5%)	2,813 (5%)	2,043 (6%)	1,210 (7%)	751 (8%)	746 (9%)
NSAID	4,889 (12%)	182 (13%)	863 (14%)	8,641 (16%)	5,647 (17%)	2,793 (16%)	1,436 (15%)	1,140 (13%)
Insulin	559 (1%)	14 (1%)	86 (1%)	942 (2%)	874 (3%)	666 (4%)	422 (5%)	394 (5%)
Other hypoglycaemics	2,985 (7%)	148 (11%)	685 (11%)	6,491 (12%)	4,897 (14%)	3,217 (18%)	1,914 (21%)	1,746 (20%)
PPI/H2 antagonists	12,745 (31%)	540 (39%)	2,769 (44%)	27,565 (50%)	19,986 (59%)	11,350 (65%)	6,565 (71%)	6,472 (76%)
**Deaths 2015-2018**								
All	2,340 (6%)	134 (10%)	454 (7%)	3,631 (7%)	3,648 (11%)	2,940 (17%)	2,286 (25%)	2,827 (33%)
Age at death	79 (70-87)	74 (66-81)	75 (67-83)	78 (70-86)	82 (74-88)	83 (76-89)	83 (76-89)	82 (74-88)
Cancer	702 (30%)	32 (24%)	136 (30%)	1,126 (31%)	991 (27%)	751 (26%)	598 (26%)	977 (35%)
GI cancer	195 (8%)	7 (5%)	36 (8%)	266 (7%)	253 (7%)	176 (6%)	132 (6%)	302 (11%)
CVD	692 (30%)	41 (31%)	122 (27%)	1,022 (28%)	1,016 (28%)	780 (27%)	598 (26%)	613 (22%)
Neuro	288 (12%)	8 (6%)	31 (7%)	448 (12%)	575 (16%)	515 (18%)	368 (16%)	355 (13%)
Chronic Resp	182 (8%)	26 (19%)	77 (17%)	408 (11%)	408 (11%)	315 (11%)	250 (11%)	277 (10%)
Infection	197 (8%)	15 (11%)	38 (8%)	272 (7%)	289 (8%)	272 (9%)	214 (9%)	282 (10%)
Other	279 (12%)	12 (9%)	50 (11%)	355 (10%)	369 (10%)	307 (10%)	258 (11%)	323 (12%)

Abbreviations – IHD: ischaemic heart disease; COPD: chronic obstructive pulmonary disease; eGFR: estimated glomerular filtration rate; GI: gastrointestinal; ECG: electrocardiogram; AF: atrial fibrillation; ACEi/ARB: angiotensin converting enzyme inhibitor/angiotensin receptor blocker; BB: beta-blocker; MRA: mineralocorticoid receptor antagonist; OAC: oral anticoagulant; NSAID: non-steroidal anti-inflammatory drugs; PPI: proton pump inhibitors; CVD: cardiovascular disease.

Presented as number and (%) or median and (Q1-Q3) for continuous variables.

Sodium-glucose Co-transporter 2 inhibitors were used in ≤1% of individuals in all categories

Blood results reported are the nadir result unless otherwise stated. The presence of any of anaemia or AF/flutter are reported.

Table S7: Characteristics of those with incident heart failure – Survivors at 31/12/2014 (all data listed on or before 31/12/2014 unless stated otherwise)								
WHO anaemia Class (Nadir 2013 to 2014)	No Hb measured	>4 above	3-4 above	1-3 above	0-1 above	0-1 below	1-2 below	>2 below
N=	72 (3%)	13 (0%)	69 (2%)	582 (21%)	622 (22%)	483 (17%)	390 (14%)	545 (20%)
Age (years) in 2013	68 (62-76)	68 (59-75)	66 (56-74)	67 (60-75)	72 (63-80)	75 (67-82)	77 (70-84)	77 (70-83)
Sex (women)	23 (32%)	7 (54%)	15 (22%)	226 (39%)	301 (48%)	252 (52%)	218 (56%)	249 (46%)
Diabetes	17 (24%)	<6 (NA%)	<6 (NA%)	107 (18%)	140 (23%)	120 (25%)	112 (29%)	175 (32%)
IHD	52 (72%)	<6 (NA%)	34 (49%)	359 (62%)	405 (65%)	316 (65%)	246 (63%)	344 (63%)
COPD	13 (18%)	6 (46%)	16 (23%)	144 (25%)	191 (31%)	134 (28%)	107 (27%)	183 (34%)
eGFR (last available)	80 (66-89)	78 (68-89)	77 (65-96)	76 (65-89)	72 (59-86)	67 (52-81)	62 (49-78)	62 (47-77)
GI disease	<6 (NA%)	0 (0%)	<6 (NA%)	28 (5%)	36 (6%)	23 (5%)	21 (5%)	70 (13%)
Any cancer prior to 2013	<6 (NA%)	0 (0%)	<6 (NA%)	31 (5%)	50 (8%)	33 (7%)	37 (9%)	59 (11%)
Any incident cancer 2013/14	0 (0%)	0 (0%)	0 (0%)	<6 (NA%)	17 (3%)	14 (3%)	18 (5%)	62 (11%)
**ECG (last result prior to 1st January 2015)**								
AF/Flutter	<6/20 (NA%)	<6/8 (NA%)	15/49 (31%)	139/408 (34%)	115/426 (27%)	104/362 (29%)	77/277 (28%)	130/442 (29%)
**Haemoglobin prior to 1st January 2013**								
Hb 4g/dL above WHO	<6 (NA%)	NA	<6 (NA%)	10 (2%)	<6 (NA%)	NA	NA	NA
Hb 3-4g/dL above WHO	<6 (NA%)	<6 (NA%)	17 (36%)	38 (8%)	11 (2%)	<6 (NA%)	<6 (NA%)	<6 (NA%)
Hb 1-3g/dL above WHO	10 (22%)	<6 (NA%)	19 (40%)	272 (60%)	202 (39%)	111 (27%)	51 (14%)	57 (12%)
Hb 0-1g/dL above WHO	16 (35%)	NA	<6 (NA%)	79 (18%)	164 (31%)	130 (31%)	79 (22%)	78 (16%)
Anaemia prior to 2013 (% of those tested)	14 (30%)	NA	<6 (NA%)	52 (12%)	139 (27%)	170 (41%)	224 (63%)	358 (72%)
**Haematinic profile (Between 2013 and 2014)**								
Ferritin (median (Q1-Q3))	298 (298-298)	113 (84-252)	164 (110-211)	128 (64-243)	88 (44-178)	72 (29-178)	58 (26-142)	45 (20-116)
Iron (median (Q1-Q3))	NA	13 (12-20)	14 (9-21)	12 (9-17)	10 (6-15)	9 (6-12)	8 (5-11)	6 (4-9)
Transferrin (median (Q1-Q3))	NA	2.2 (1.9-2.8)	2.4 (2.1-3.0)	2.6 (2.3-2.8)	2.3 (2.0-2.6)	2.3 (2.0-2.7)	2.3 (2.0-2.7)	2.4 (2.0-3.0)
TSAT (median (Q1-Q3))	NA	27 (23-29)	27 (17-32)	18 (13-29)	18 (11-24)	16 (11-21)	13 (9-20)	10 (6-17)
B12 (median (Q1-Q3))	NA	412 (378-760)	368 (222-439)	338 (263-452)	361 (274-477)	352 (258-448)	328 (248-422)	331 (226-464)
Folate (median (Q1-Q3))	NA	4.1 (3.0-4.8)	3.1 (2.6-4.3)	5.0 (3.3-6.9)	4.3 (2.8-6.6)	4.8 (3.3-7.3)	4.3 (2.9-6.7)	4.1 (2.9-6.3)

Serum sodium	138 (137-140)	138 (135-140)	137 (135-138)	137 (135-139)	136 (134-138)	135 (133-138)	135 (132-137)	135 (131-137)
Albumin	38 (36 - 39)	35 (33 - 37)	35 (34 - 37)	36 (33 - 38)	34 (32 - 36)	33 (31 - 35)	32 (29 - 34)	30 (27 - 33)
**Prescriptions (on 1st January 2015)**								
Iron (oral)	<6 (NA%)	<6 (NA%)	0 (0%)	11 (2%)	17 (3%)	47 (10%)	77 (20%)	226 (41%)
B12	<6 (NA%)	0 (0%)	0 (0%)	8 (1%)	23 (4%)	21 (4%)	19 (5%)	44 (8%)
Folate	<6 (NA%)	<6 (NA%)	<6 (NA%)	20 (3%)	48 (8%)	42 (9%)	63 (16%)	98 (18%)
Loop diuretics	15 (21%)	6 (46%)	31 (45%)	285 (49%)	333 (54%)	308 (64%)	270 (69%)	399 (73%)
ACEi/ARB	44 (61%)	7 (54%)	48 (70%)	451 (77%)	447 (72%)	337 (70%)	265 (68%)	338 (62%)
BB	44 (61%)	<6 (NA%)	47 (68%)	423 (73%)	415 (67%)	332 (69%)	245 (63%)	349 (64%)
MRA	<6 (NA%)	<6 (NA%)	9 (13%)	88 (15%)	86 (14%)	90 (19%)	63 (16%)	97 (18%)
Antiplatelets	40 (56%)	6 (46%)	39 (57%)	336 (58%)	397 (64%)	300 (62%)	255 (65%)	331 (61%)
OAC	11 (15%)	<6 (NA%)	21 (30%)	201 (35%)	180 (29%)	143 (30%)	110 (28%)	150 (28%)
NSAID	7 (10%)	<6 (NA%)	7 (10%)	31 (5%)	41 (7%)	28 (6%)	19 (5%)	28 (5%)
Insulin	<6 (NA%)	0 (0%)	0 (0%)	10 (2%)	17 (3%)	21 (4%)	20 (5%)	33 (6%)
Other hypoglycaemics	9 (12%)	<6 (NA%)	<6 (NA%)	55 (9%)	96 (15%)	88 (18%)	71 (18%)	116 (21%)
PPI/H2 antagonists	26 (36%)	<6 (NA%)	26 (38%)	318 (55%)	374 (60%)	322 (67%)	305 (78%)	423 (78%)
**Deaths 2015-2018**								
All	7 (10%)	<6 (NA%)	8 (12%)	88 (15%)	147 (24%)	162 (34%)	160 (41%)	259 (48%)
Age at death	84 (83-90)	80 (76-86)	77 (74-85)	80 (73-86)	82 (75-89)	84 (77-89)	86 (79-91)	85 (79-89)
Cancer	<6 (NA%)	0 (0%)	<6 (NA%)	16 (18%)	22 (15%)	25 (15%)	24 (15%)	46 (18%)
GI cancer	0 (0%)	NA	<6 (NA%)	<6 (NA%)	<6 (NA%)	<6 (NA%)	7 (4%)	14 (5%)
CVD	<6 (NA%)	<6 (NA%)	<6 (NA%)	33 (38%)	56 (38%)	65 (40%)	58 (36%)	105 (41%)
Neuro	<6 (NA%)	0 (0%)	<6 (NA%)	9 (10%)	18 (12%)	9 (6%)	22 (14%)	24 (9%)
Chronic Resp	0 (0%)	0 (0%)	<6 (NA%)	20 (23%)	31 (21%)	27 (17%)	23 (14%)	34 (13%)
Infection	0 (0%)	0 (0%)	0 (0%)	<6 (NA%)	7 (5%)	18 (11%)	15 (9%)	28 (11%)
Other	0 (0%)	0 (0%)	0 (0%)	6 (7%)	13 (10%)	18 (11%)	18 (11%)	22 (8%)

Abbreviations – IHD: ischaemic heart disease; COPD: chronic obstructive pulmonary disease; eGFR: estimated glomerular filtration rate; GI: gastrointestinal; ECG: electrocardiogram; AF: atrial fibrillation; ACEi/ARB: angiotensin converting enzyme inhibitor/angiotensin receptor blocker; BB: beta-blocker; MRA:

mineralocorticoid receptor antagonist; OAC: oral anticoagulant; NSAID: non-steroidal anti-inflammatory drugs; PPI: proton pump inhibitors; CVD: cardiovascular disease.

Presented as number and (%) or median and (Q1-Q3) for continuous variables.

Sodium-glucose Co-transporter 2 inhibitors were used in ≤1% of individuals in all categories

Blood results reported are the nadir result unless otherwise stated. The presence of any of anaemia or AF/flutter are reported.

**Table S8: Characteristics of those with prevalent heart failure – Survivors at 31/12/2014 (all data listed on or before 31/12/2014 unless stated otherwise)**								
WHO anaemia Class (Nadir 2013 to 2014)	No Hb measured	>4 above	3-4 above	1-3 above	0-1 above	0-1 below	1-2 below	>2 below
N=	1,093 (12%)	61 (1%)	261 (3%)	2,315 (26%)	1,904 (21%)	1,402 (16%)	901 (10%)	962 (11%)
Age (years) in 2013	68 (61-76)	62 (56-70)	66 (59-74)	68 (61-76)	73 (65-80)	76 (68-81)	77 (69-82)	77 (70-83)
Sex (women)	350 (32%)	6 (10%)	68 (26%)	777 (34%)	857 (45%)	643 (46%)	402 (45%)	375 (39%)
Diabetes	216 (20%)	11 (18%)	63 (24%)	569 (25%)	461 (24%)	403 (29%)	286 (32%)	344 (36%)
IHD	747 (68%)	39 (64%)	200 (77%)	1,746 (75%)	1,481 (78%)	1,052 (75%)	695 (77%)	749 (78%)
COPD	172 (16%)	14 (23%)	68 (26%)	519 (22%)	526 (28%)	393 (28%)	274 (30%)	314 (33%)
eGFR (last available)	78 (65-91)	83 (69-94)	78 (64-90)	76 (64-90)	71 (57-85)	69 (53-84)	64 (49-79)	62 (47-77)
GI disease	37 (3%)	<6 (NA%)	8 (3%)	89 (4%)	117 (6%)	84 (6%)	88 (10%)	122 (13%)
Any cancer prior to 2013	55 (5%)	<6 (NA%)	15 (6%)	138 (6%)	184 (10%)	130 (9%)	116 (13%)	113 (12%)
Any incident cancer 2013/14	<6 (NA%)	<6 (NA%)	<6 (NA%)	25 (1%)	30 (2%)	41 (3%)	46 (5%)	69 (7%)
**ECG (last result prior to 1st January 2015)**								
AF/Flutter	41/204 (20%)	<6/16 (NA%)	23/79 (29%)	222/918 (24%)	185/867 (21%)	167/742 (23%)	142/537 (26%)	177/647 (27%)
**Haemoglobin prior to 1st January 2013**								
Hb 4g/dL above WHO	17 (2%)	14 (26%)	7 (3%)	10 (0%)	<6 (NA%)	<6 (NA%)	NA	<6 (NA%)
Hb 3-4g/dL above WHO	35 (4%)	20 (38%)	50 (21%)	67 (3%)	11 (1%)	<6 (NA%)	<6 (NA%)	<6 (NA%)
Hb 1-3g/dL above WHO	327 (41%)	11 (21%)	132 (55%)	1,198 (56%)	356 (20%)	121 (9%)	48 (6%)	36 (4%)
Hb 0-1g/dL above WHO	178 (22%)	<6 (NA%)	29 (12%)	508 (24%)	740 (41%)	346 (26%)	134 (15%)	72 (8%)
Anaemia prior to 2013 (% of those tested)	241 (30%)	6 (11%)	20 (8%)	365 (17%)	706 (39%)	870 (65%)	684 (79%)	822 (88%)
**Haematinic profile (Between 2013 and 2014)**								
Ferritin (median (Q1-Q3))	128 (87-246)	146 (62-208)	122 (72-249)	109 (57-220)	85 (40-164)	72 (31-152)	54 (24-131)	43 (16-112)
Iron (median (Q1-Q3))	18 (15-22)	NA	18 (14-23)	14 (10-20)	12 (9-16)	12 (8-16)	9 (6-13)	6 (4-10)
Transferrin (median (Q1-Q3))	2.1 (1.9-2.3)	NA	2.5 (2.2-2.7)	2.5 (2.0-2.8)	2.4 (2.0-2.7)	2.3 (2.0-2.7)	2.4 (2.0-2.8)	2.3 (1.8-2.8)
TSAT (median Q1-Q3))	33 (29-37)	NA	23 (23-36)	23 (17-33)	20 (15-26)	19 (13-27)	16 (10-23)	11 (6-19)
B12 (median (Q1-Q3))	285 (192-365)	372 (276-476)	350 (282-408)	334 (255-447)	322 (245-436)	330 (244-452)	316 (242-461)	332 (237-460)
Folate (median (Q1-Q3))	8.8 (5.4-11.0)	4.2 (2.7-5.6)	4.0 (3.0-5.8)	4.6 (3.3-6.8)	4.6 (3.2-7.2)	4.4 (3.2-7.0)	4.6 (3.2-7.0)	4.5 (3.1-7.2)

Serum sodium	139 (137-140)	138 (136-139)	138 (136-140)	138 (136-139)	137 (135-139)	136 (134-38)	136 (133-138)	135 (132-137)
Albumin	37 (36-39)	37 (35-39)	37 (35-39)	37 (35-38)	36 (33-37)	34 (32-37)	33 (30-35)	31 (28-34)
**Prescriptions (on 1st January 2015)**								
Iron (oral)	25 (2%)	0 (0%)	<6 (NA%)	40 (2%)	77 (4%)	126 (9%)	166 (18%)	380 (40%)
B12	15 (1%)	<6 (NA%)	<6 (NA%)	43 (2%)	66 (3%)	78 (6%)	62 (7%)	90 (9%)
Folate	26 (2%)	<6 (NA%)	8 (3%)	95 (4%)	139 (7%)	150 (11%)	122 (14%)	223 (23%)
Loop diuretics	345 (32%)	24 (39%)	94 (36%)	945 (41%)	899 (47%)	757 (54%)	521 (58%)	640 (67%)
ACEi/ARB	818 (75%)	49 (80%)	200 (77%)	1,837 (79%)	1,483 (78%)	1,051 (75%)	609 (68%)	616 (64%)
BB	747 (68%)	42 (69%)	199 (76%)	1,645 (71%)	1,316 (69%)	891 (64%)	591 (66%)	574 (60%)
MRA	73 (7%)	6 (10%)	28 (11%)	269 (12%)	216 (11%)	201 (14%)	115 (13%)	129 (13%)
Antiplatelets	684 (63%)	33 (54%)	170 (65%)	1,482 (64%)	1,289 (68%)	887 (63%)	575 (64%)	572 (59%)
OAC	192 (18%)	19 (31%)	81 (31%)	602 (26%)	456 (24%)	375 (27%)	245 (27%)	278 (29%)
NSAID	40 (4%)	<6 (NA%)	10 (4%)	134 (6%)	126 (7%)	89 (6%)	54 (6%)	57 (6%)
Insulin	29 (3%)	<6 (NA%)	8 (3%)	71 (3%)	80 (4%)	76 (5%)	66 (7%)	76 (8%)
Other hypoglycaemics	132 (12%)	7 (11%)	35 (13%)	345 (15%)	299 (16%)	286 (20%)	172 (19%)	241 (25%)
PPI/H2 antagonists	427 (39%)	31 (51%)	119 (46%)	1,294 (56%)	1,210 (64%)	955 (68%)	653 (72%)	758 (79%)
**Deaths 2015-2018**								
All	142 (13%)	7 (11%)	32 (12%)	345 (15%)	401 (21%)	376 (27%)	295 (33%)	432 (45%)
Age at death	84 (74 - 89)	59 (57 - 60)	74 (67 - 82)	81 (74 - 88)	83 (77 - 90)	84 (78 - 90)	84 (77 - 90)	83 (77 - 89)
Cancer	22 (15%)	<6 (NA%)	<6 (NA%)	72 (21%)	60 (15%)	55 (15%)	47 (16%)	85 (20%)
GI cancer	8 (6%)	0 (0%)	<6 (NA%)	14 (4%)	14 (3%)	12 (3%)	14 (5%)	27 (6%)
CVD	51 (36%)	<6 (NA%)	15 (47%)	129 (37%)	164 (41%)	155 (41%)	129 (44%)	149 (34%)
Neuro	22 (15%)	0 (0%)	6 (19%)	38 (11%)	47 (12%)	39 (10%)	43 (15%)	37 (9%)
Chronic Resp	15 (11%)	0 (0%)	<6 (NA%)	47 (14%)	65 (16%)	48 (13%)	32 (11%)	55 (13%)
Infection	15 (11%)	0 (0%)	<6 (NA%)	28 (8%)	37 (9%)	32 (9%)	24 (8%)	48 (11%)
Other	17 (12%)	<6 (NA%)	<6 (NA%)	31 (9%)	28 (7%)	47 (12%)	20 (7%)	58 (14%)

Abbreviations – IHD: ischaemic heart disease; COPD: chronic obstructive pulmonary disease; eGFR: estimated glomerular filtration rate; GI: gastrointestinal; ECG: electrocardiogram; AF: atrial fibrillation; ACEi/ARB: angiotensin converting enzyme inhibitor/angiotensin receptor blocker; BB: beta-blocker; MRA:

mineralocorticoid receptor antagonist; OAC: oral anticoagulant; NSAID: non-steroidal anti-inflammatory drugs; PPI: proton pump inhibitors; CVD: cardiovascular disease.

Presented as number and (%) or median and (Q1-Q3) for continuous variables.

Sodium-glucose Co-transporter 2 inhibitors were used in ≤1% of individuals in all categories

Blood results reported are the nadir result unless otherwise stated. The presence of any of anaemia or AF/flutter are reported.

**Table S9: Number and % of patients with ferritin test by haemoglobin concentration according to age and sex**									
Hb concentration By W.H.O grade	Sex	<60 years	60-70 years	P values: sex	70-80 years	>80 years	P values: sex	P values: age groups	Total number of patients
>1g/dL above	Female	754 (6%)	630 (6%)	p<0.001	560 (7%)	436 (11%)	p<0.001	p<0.001	34,907
	Male	592 (5%)	496 (4%)		349 (5%)	131 (7%)			33,008
0-1g/dL above	Female	559 (9%)	568 (9%)	P<0.001	766 (12%)	614 (14%)	p<0.001	p<0.001	23,059
	Male	327 (8%)	391 (8%)		347 (8%)	210 (11%)			15,236
0-1g/dL below	Female	487 (21%)	607 (22%)	p=0.003	958 (24%)	913 (24%)	p=0.784	p<0.007	12,933
	Male	360 (25%)	566 (24%)		687 (25%)	388 (23%)			8,294
1-2g/dL below	Female	388 (40%)	605 (42%)	p<0.001	1,014 (45%)	1,114 (44%)	P<0.001	p=0.01	7,213
	Male	266 (35%)	432 (33%)		632 (35%)	462 (34%)			5,218
>2g/dL below	Female	424 (49%)	668 (52%)	p=0.019	1,188 (55%)	1,327 (56%)	p<0.001	p=0.005	6,695
	Male	416 (46%)	775 (48%)		1,135 (46%)	967 (49%)			6,946

Abbreviations:- Hb: haemoglobin

In comparing differences in testing patterns between age groups (<70 years vs ≥70 years), the total number of patients in each age group, irrespective of sex, were compared.

In comparing differences in testing patterns between patients' sex, age groups were combined to compare testing patterns in those <70 years and those ≥70 years.

Table S10: Number and % of patients with TSAT test by haemoglobin concentration according to age and sex

Hb concentration By W.H.O grade	Sex	<60 years	60-70 years	P values: sex	70-80 years	>80 years	P values: sex	P values: age groups	Total number of patients
>1g/dL above	Female	157 (1%)	144 (1%)	p = 1	111 (1%)	112 (3%)	P=0.39	p<0.001	34,907
	Male	178 (1%)	152 (1%)		91 (1%)	44 (2%)			33,008
0-1g/dL above	Female	104 (2%)	113 (2%)	p=0.002	156 (2%)	173 (4%)	p=0.006	p<0.001	23,059
	Male	107 (3%)	112 (2%)		84 (2%)	57 (3%)			15,236
0-1g/dL below	Female	91 (4%)	137 (5%)	p<0.001	225 (6%)	239 (6%)	p=0.75	p<0.001	12,933
	Male	98 (7%)	142 (6%)		165 (6%)	106 (6%)			8,294
1-2g/dL below	Female	111 (12%)	161 (11%)	p=1	294 (13%)	403 (16%)	P<0.001	p=0.01	7,213
	Male	82 (11%)	154 (12%)		188 (11%)	152 (11%)			5,218
>2g/dL below	Female	172 (20%)	265 (21%)	p=0.838	434 (20%)	556 (23%)	p=0.13	p=0.39	6,695
	Male	195 (22%)	324 (20%)		472 (19%)	434 (22%)			6,946

Abbreviations:- Hb: haemoglobin

In comparing differences in testing patterns between age groups (<70 years vs ≥70 years), the total number of patients in each age group, irrespective of sex, were compared.

In comparing differences in testing patterns between patients' sex, age groups were combined to compare testing patterns in those <70 years and those ≥70 years.

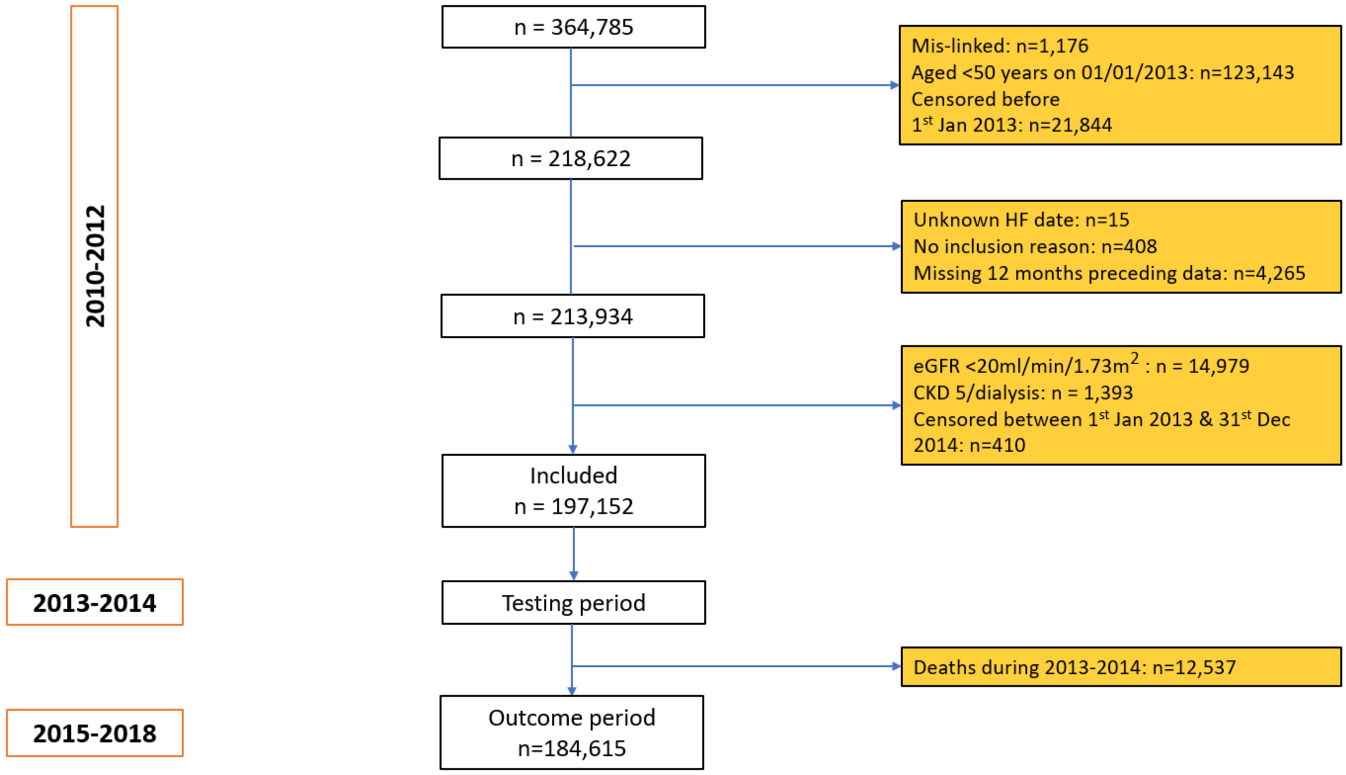

Figure S1: STROBE diagram of included participants. Exclusions with number and (%) of each are presented in boxes on the right-hand panel. Panels on the left indicate the year(s) of each part of the study.

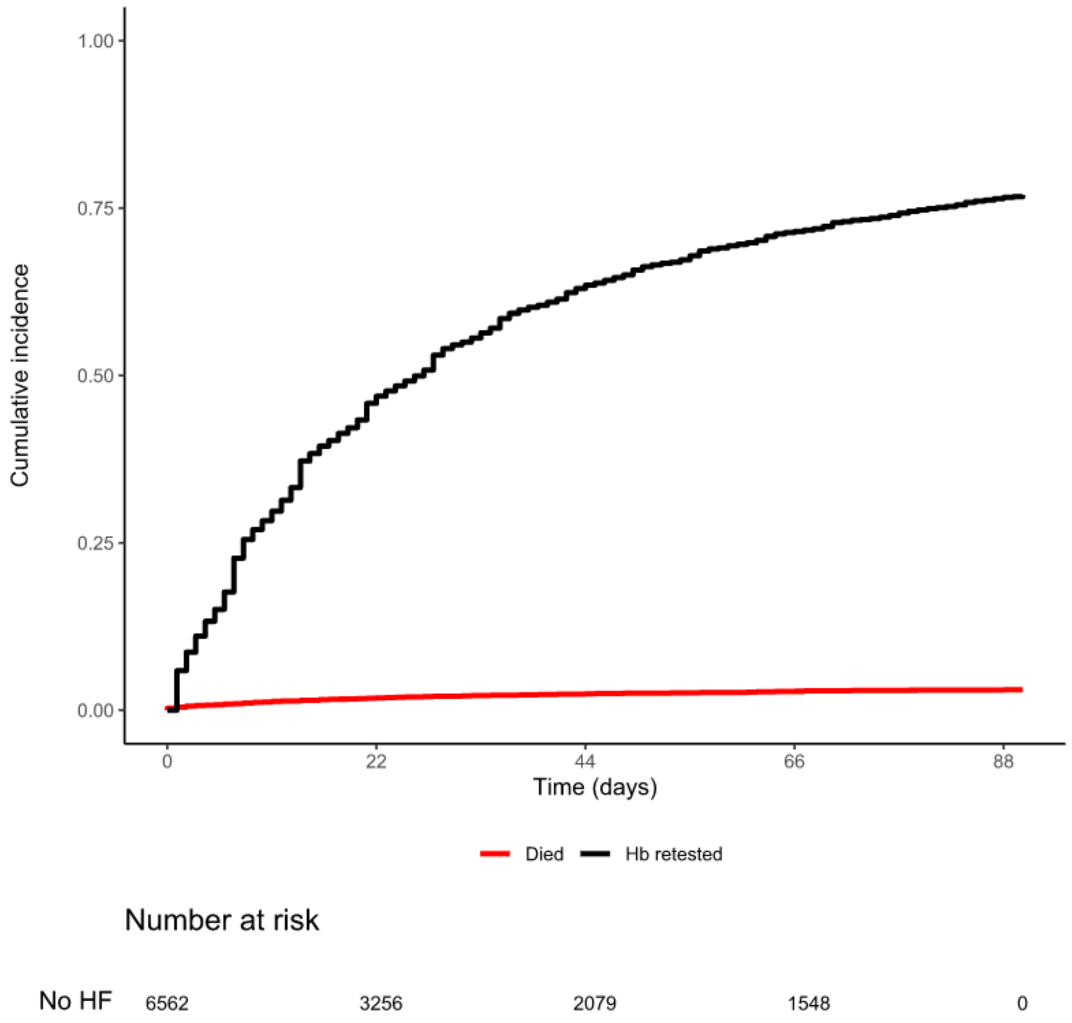

Figure S2: Cumulative incidence plots of rates of re-testing of haemoglobin (black) and all-cause mortality (red) following a diagnosis of severe anaemia (<2g/dL below W.H.O.) between 2013/14 in those without a history of heart failure.

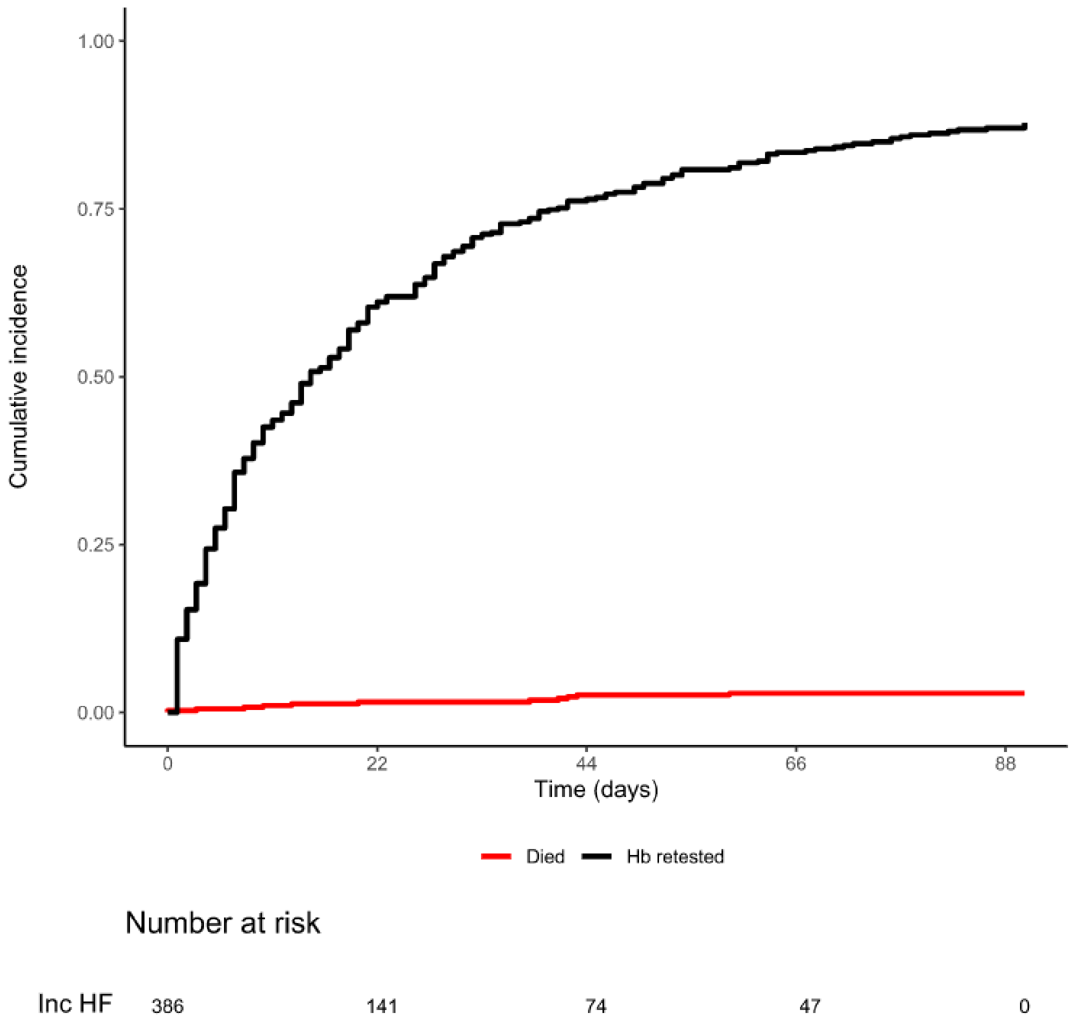

Figure S3: Cumulative incidence plots of rates of re-testing of haemoglobin (black) and all-cause mortality (red) following a diagnosis of severe anaemia (<2g/dL below W.H.O.) between 2013/14 in those with incident heart failure.

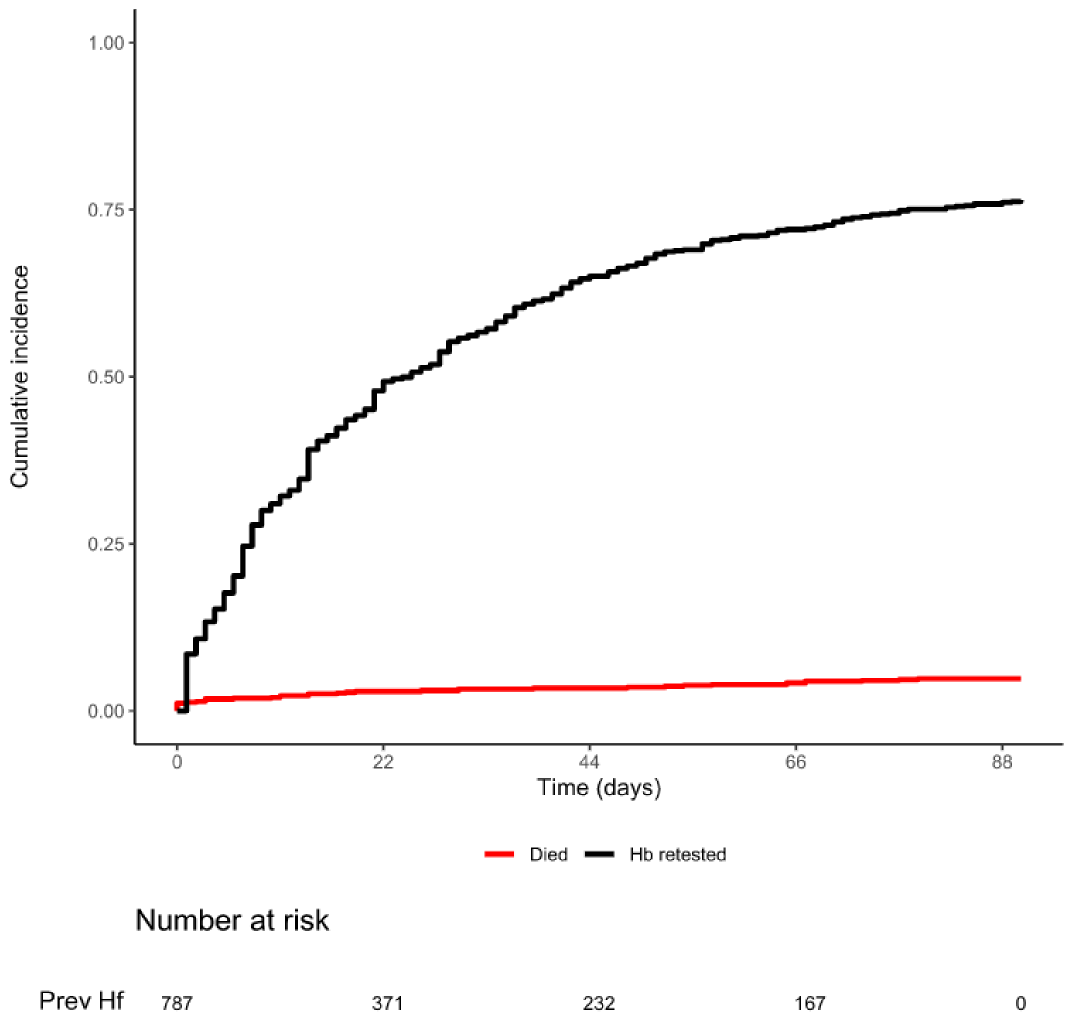

Figure S4: Cumulative incidence plots of rates of re-testing of haemoglobin (black) and all-cause mortality (red) following a diagnosis of severe anaemia (<2g/dL below W.H.O.) between 2013/14 in those with prevalent heart failure.

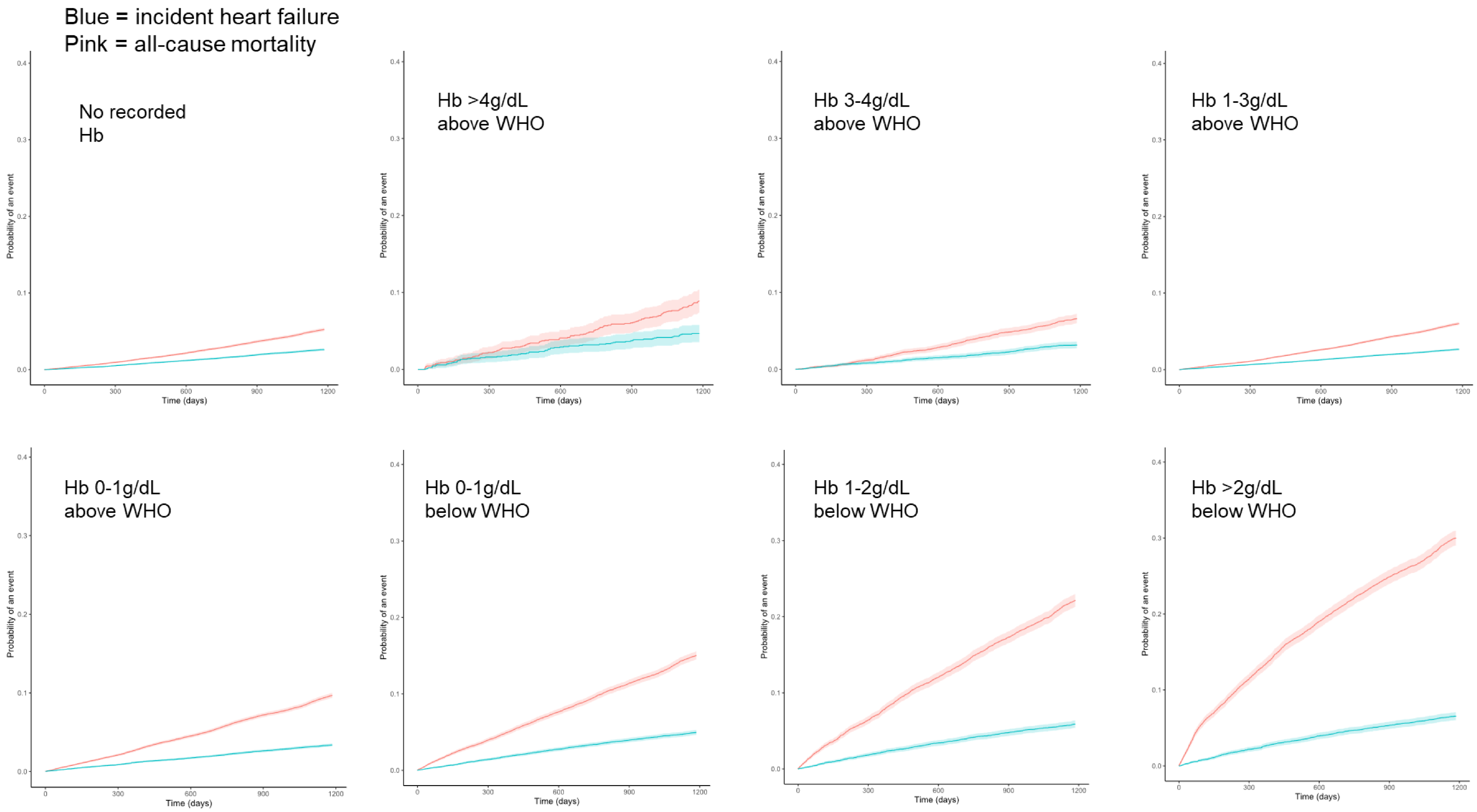

Figure S5: Cumulative incidence curves of incident heart failure (blue) and all-cause mortality (pink). Associated 95% Confidence Intervals from 1st January 2015 to 31st March 2018 by haemoglobin concentration for patients who survived, free of heart failure up to 31st December 2014. Hb: haemoglobin; W.H.O.: World Health Organization

A

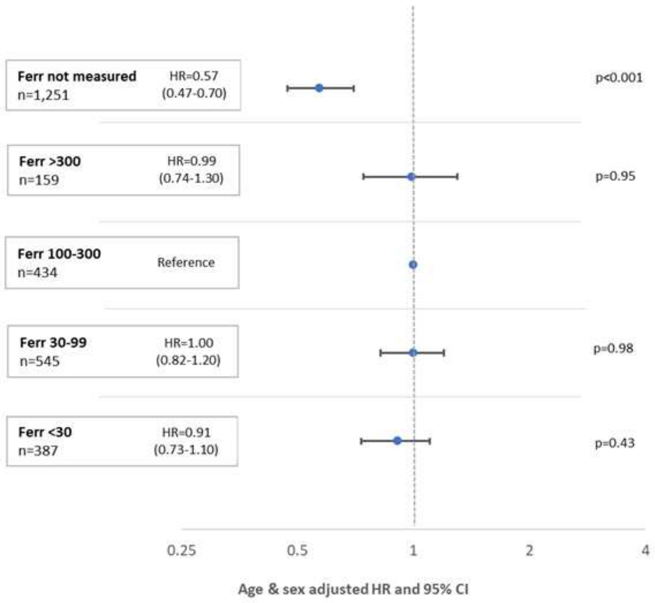
Ferr not measured
n=1,251
HR=0.57
(0.47-0.70)
p<0.001
Ferr >300
n=159
HR=0.99
(0.74-1.30)
p=0.95
Ferr 100-300
n=434
Reference
Ferr 30-99
n=545
HR=1.00
(0.82-1.20)
p=0.98
Ferr <30
n=387
HR=0.91
(0.73-1.10)
p=0.43
0.25
0.5
1
2
4
Age & sex adjusted HR and 95% CI

B

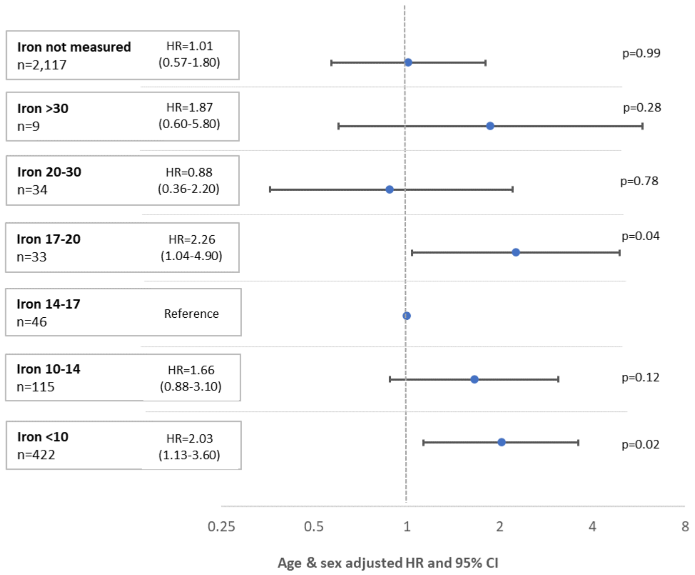
Iron not measured
n=2,117
HR=1.01
(0.57-1.80)
p=0.99
Iron >30
n=9
HR=1.87
(0.60-5.80)
p=0.28
Iron 20-30
n=34
HR=0.88
(0.36-2.20)
p=0.78
Iron 17-20
n=33
HR=2.26
(1.04-4.90)
p=0.04
Iron 14-17
n=46
Reference
Iron 10-14
n=115
HR=1.66
(0.88-3.10)
p=0.12
Iron <10
n=422
HR=2.03
(1.13-3.60)
p=0.02
0.25
0.5
1
2
4
8
Age & sex adjusted HR and 95% CI

C

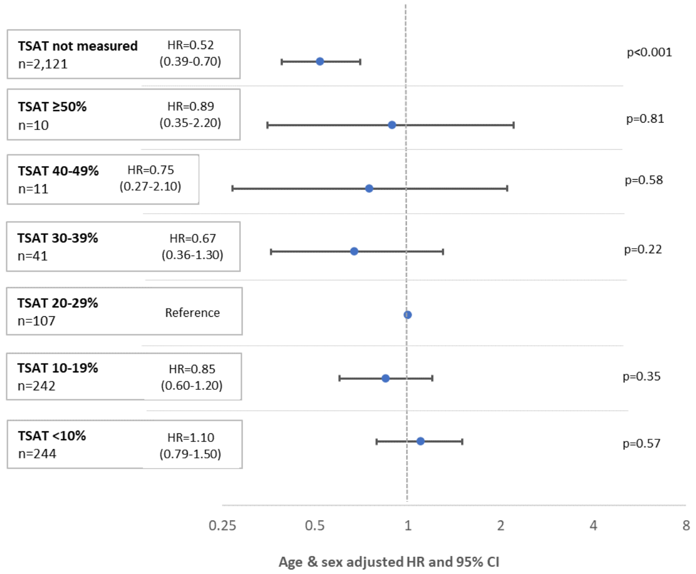

Figure S6: Forest plots showing all-cause mortality from 1st January 2015 to 31st March 2018 in surviving patients with incident heart failure during 2013/14 according to concentrations of serum ferritin (A) (Not measured; >300 µg/L; 100-300 µg/L; 30-100 µg/L; <30 µg/L), serum iron (B) (Not measured; >30 µmol/L; 20-30 µmol/L; 17-20 µmol/L; 14-17 µmol/L; 10-14 µmol/L; <10 µmol/L) and transferrin saturation (TSAT) (C) (Not measured; ≥50%; 40-49%; 30-39%; 20-29%; 10-19%; <10%).

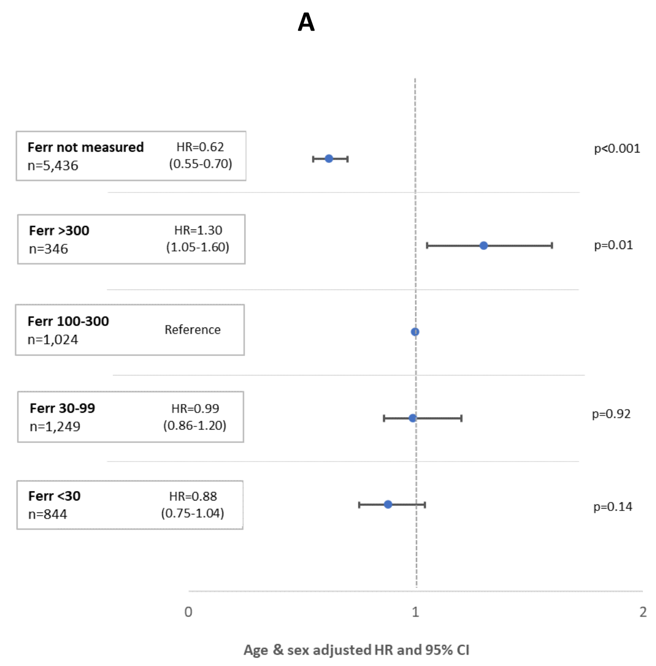
A
Ferr not measured n=5,436 HR=0.62 (0.55-0.70) p<0.001
Ferr >300 n=346 HR=1.30 (1.05-1.60) p=0.01
Ferr 100-300 n=1,024 Reference
Ferr 30-99 n=1,249 HR=0.99 (0.86-1.20) p=0.92
Ferr <30 n=844 HR=0.88 (0.75-1.04) p=0.14
0
1
2
Age & sex adjusted HR and 95% CI

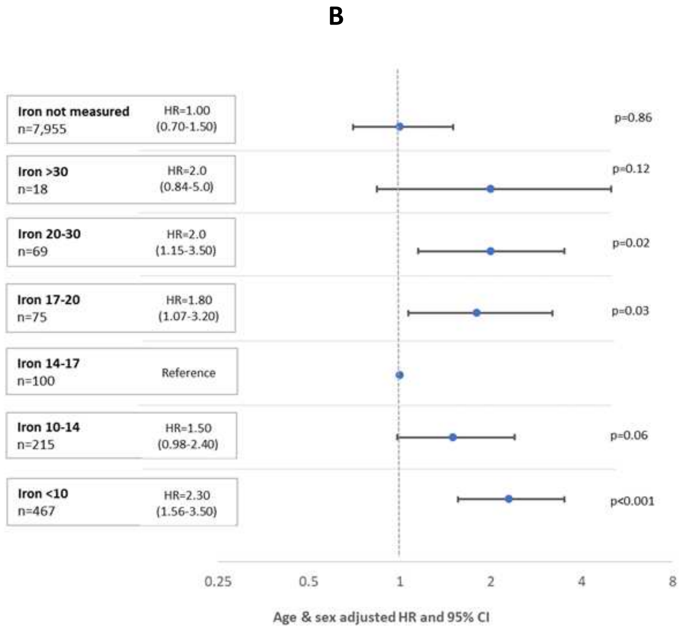
B
Iron not measured n=7,955 HR=1.00 (0.70-1.50) p=0.86
Iron >30 n=18 HR=2.0 (0.84-5.0) p=0.12
Iron 20-30 n=69 HR=2.0 (1.15-3.50) p=0.02
Iron 17-20 n=75 HR=1.80 (1.07-3.20) p=0.03
Iron 14-17 n=100 Reference
Iron 10-14 n=215 HR=1.50 (0.98-2.40) p=0.06
Iron <10 n=467 HR=2.30 (1.56-3.50) p<0.001
0.25
0.5
1
2
4
8
Age & sex adjusted HR and 95% CI

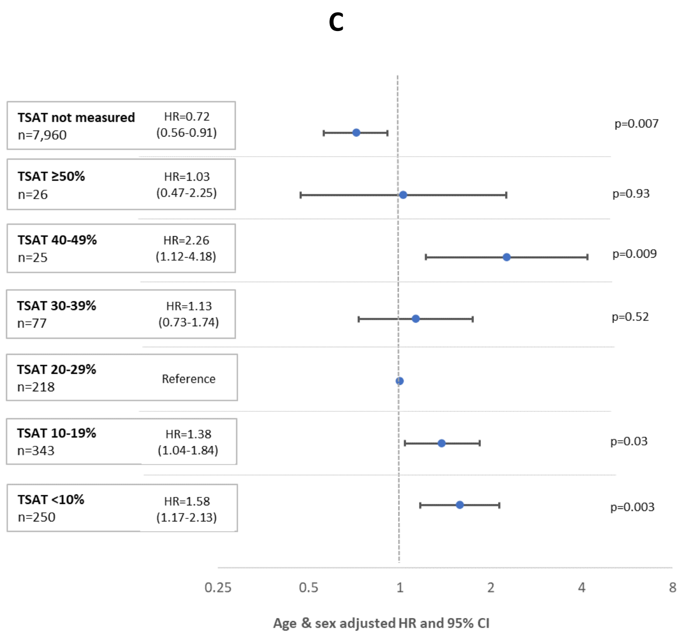

Figure S7: Forest plots showing all-cause mortality from 1st January 2015 to 31st March 2018 in surviving patients with prevalent heart failure during 2013/14 according to concentrations of serum ferritin (A) (Not measured; >300 µg/L; 100-300 µg/L; 30-100 µg/L; <30 µg/L), serum iron (B) (Not measured; >30 µmol/L; 20-30 µmol/L; 17-20 µmol/L; 14-17 µmol/L; 10-14 µmol/L; <10 µmol/L) and transferrin saturation (TSAT) (C) (Not measured; ≥50%; 40-49%; 30-39%; 20-29%; 10-19%; <10%).

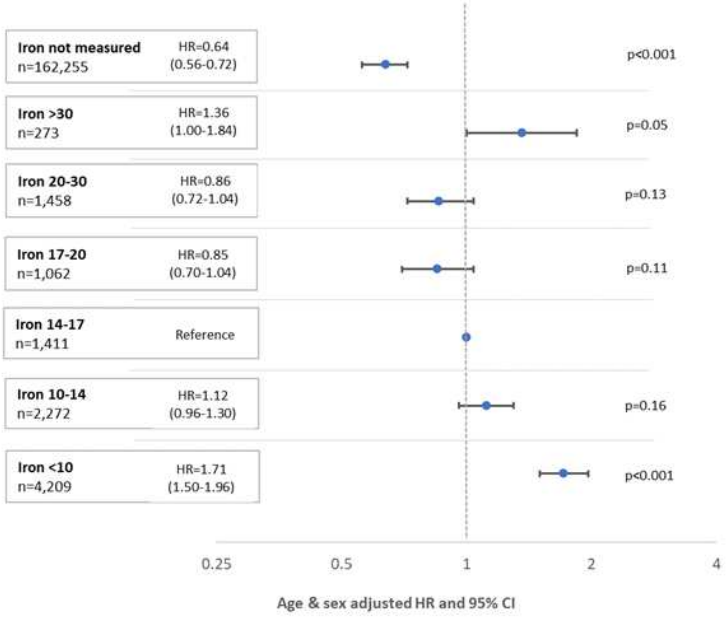

Figure S8: Forest plots showing all-cause mortality from 1st January 2015 to 31st March 2018 in surviving patients without a history of heart failure during, or prior to, 2013/14 according to concentrations of serum iron (Not measured; >30 µmol/L; 20-30 µmol/L; 17-20 µmol/L; 14-17 µmol/L; 10-14 µmol/L; <10 µmol/L).
